# Unusual nitrene reactivity: imine formation in the photochemical reaction of a borylnitrene with ethene

**DOI:** 10.1039/d5sc07994b

**Published:** 2025-12-24

**Authors:** Virinder Bhagat, Holger F. Bettinger

**Affiliations:** a Institut für Organische Chemie, Universität Tübingen Auf der Morgenstelle 18 72076 Tübingen Germany holger.bettinger@uni-tuebingen.de

## Abstract

The cycloaddition of nitrenes with olefins is an important method for the synthesis of aziridines. We report here that the reaction of catecholato borylnitrene CatBN with ethene upon long wavelength photoirradiation (*λ* > 550 nm) gives not only the expected aziridine, but also the imine CatBN=CHCH_3_ under cryogenic matrix isolation conditions in solid neon at 4 K. Computational analysis reveals that the photochemically generated singlet CatBN (^1^A_1_ electronic state) can react with ethene to form the aziridine and a singlet 1,3-diradical intermediate. The latter arises from multi-state reactivity involving the nearly degenerate ^1^A_1_ and ^1^A_2_ nitrene singlet states. The singlet 1,3-diradical has a very low barrier for 1,2-H migration to give the imine. The observation of an imine in the reaction with an alkene reveals chemodivergence by highlighting a previously unobserved reactivity pathway in nitrene chemistry.

## Abstract 2

Catecholato borylnitrene reacts with ethene to produce aziridine and an unexpected isomeric imine, formed *via* a singlet 1,3-diradical pathway revealed under cryogenic matrix conditions.

## Graphical abstract

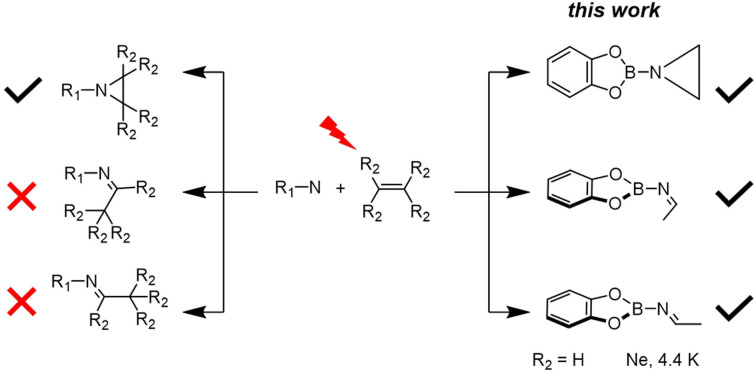

## Introduction

**Scheme 1:**
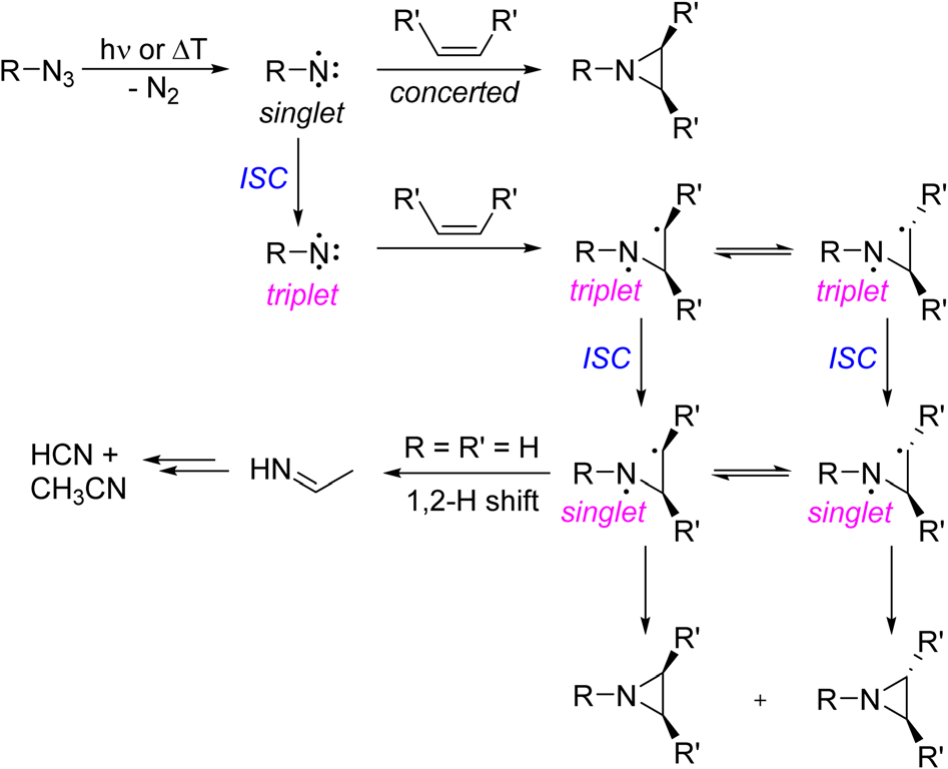
Simplified reaction scheme of photochemically or thermally generated nitrenes with olefins. Aziridines are typically observed in solution, while in the gas phase fragmentation occurs in the reaction of triplet NH with ethene (R=R′=H).

The reaction of nitrenoids and free nitrenes with carbon–carbon double bonds is an important method for producing aziridines in organic synthesis.^1–10^ The hallmark of singlet nitrenes R–N, electron deficient nitrogen species with an electron sextet, is their stereospecific cycloaddition reaction with olefins to yield aziridines.^2^ The cycloaddition of singlet nitrenes with alkenes is a concerted reaction that preserves the stereochemistry of the alkene substituents (Scheme 1). In contrast, triplet nitrenes react in a stepwise fashion involving a triplet 1,3-diradical intermediate. As the rate of intersystem crossing to the lower energy singlet 1,3-diradical can be competitive with the rate of rotation about the C–C bond of the diradical, triplet nitrenes show only low or even no stereoselectivity in aziridine formation.^2^

In the gas phase reaction of imidogen NH and ethene, the primary products, aziridine and triplet 1,3-diradical, are formed in a vibrationally hot state and fragment to the ultimate stable products CH_3_CN and HCN (Scheme 1, bottom).^11–14^ Vibrationally excited aziridine is assumed to be in dynamic equilibrium with the singlet 1,3-diradical that can readily rearrange to an imine by 1,2-hydrogen shift.^13,15^ The imine is assumed to be an intermediate of gas phase product formation, but has been rarely observed in the reaction of nitrenes with olefins.^8^

**Scheme 2:**
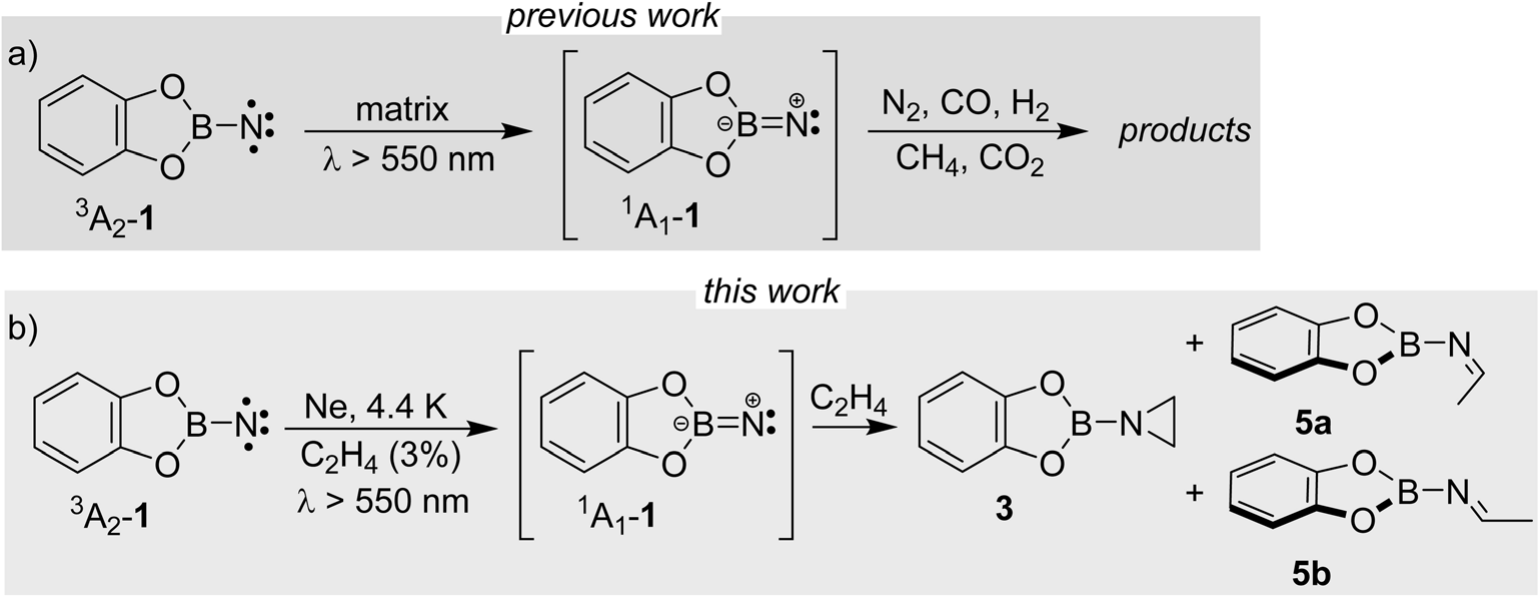
(a) Photochemical reaction of triplet borylnitrene 1 with the closed-shell molecules; (b) products observed after photolysis of ^3^A_2_-1 to ^1^A_1_-1 in the presence of ethene under matrix isolation conditions.

We here report detection of an imine in the reaction of a singlet borylnitrene with ethene. Borylnitrenes are a rather unusual class of nitrenes. The catecholato borylnitrene CatBN 1, a triplet ground state species (^3^A_2_ electronic state) that can be observed using matrix isolation methods,^16^ reacts upon very mild annealing with dioxygen O_2_ to form nitritoborane CatBONO revealing that ^3^A_2_-1 is much more reactive than typical organic nitrenes.^16,17^ Upon long wavelength irradiation (>550 nm), matrix isolated ^3^A_2_-1 forms ^1^A_1_-1 as supported by computational analysis of the excited state potential energy surfaces.^18^ The highly electrophilic vinylidene-like ^1^A_1_-1 readily activates unreactive small closed-shell molecules by addition (N_2_, CO, and CO_2_) or insertion (CH_4_ and H_2_) into single bonds (Scheme 2a).^16,19–23^ The triplet state of 1 does not react with these unreactive species. In solution or the gas phase, borylnitrenes of the 1,3,2-dioxaborolane type insert into unactivated CH bonds allowing for amination of unusual substrates, such as cycloalkanes, methane, and tetramethylsilane presumably *via* the transient singlet nitrene.^19,21,24^

By combining matrix isolation IR spectroscopy and computational methods, we provide evidence that the reaction of CatBN with ethene yields, along with the expected aziridine, the isomeric imine, demonstrating chemodivergence^25^ in this simple system (Scheme 2b). The imine likely results from the singlet 1,3-diradical, which in turn arises from the multi-state reactivity of the borylnitrene.

## Results and discussion

**Fig. 1:**
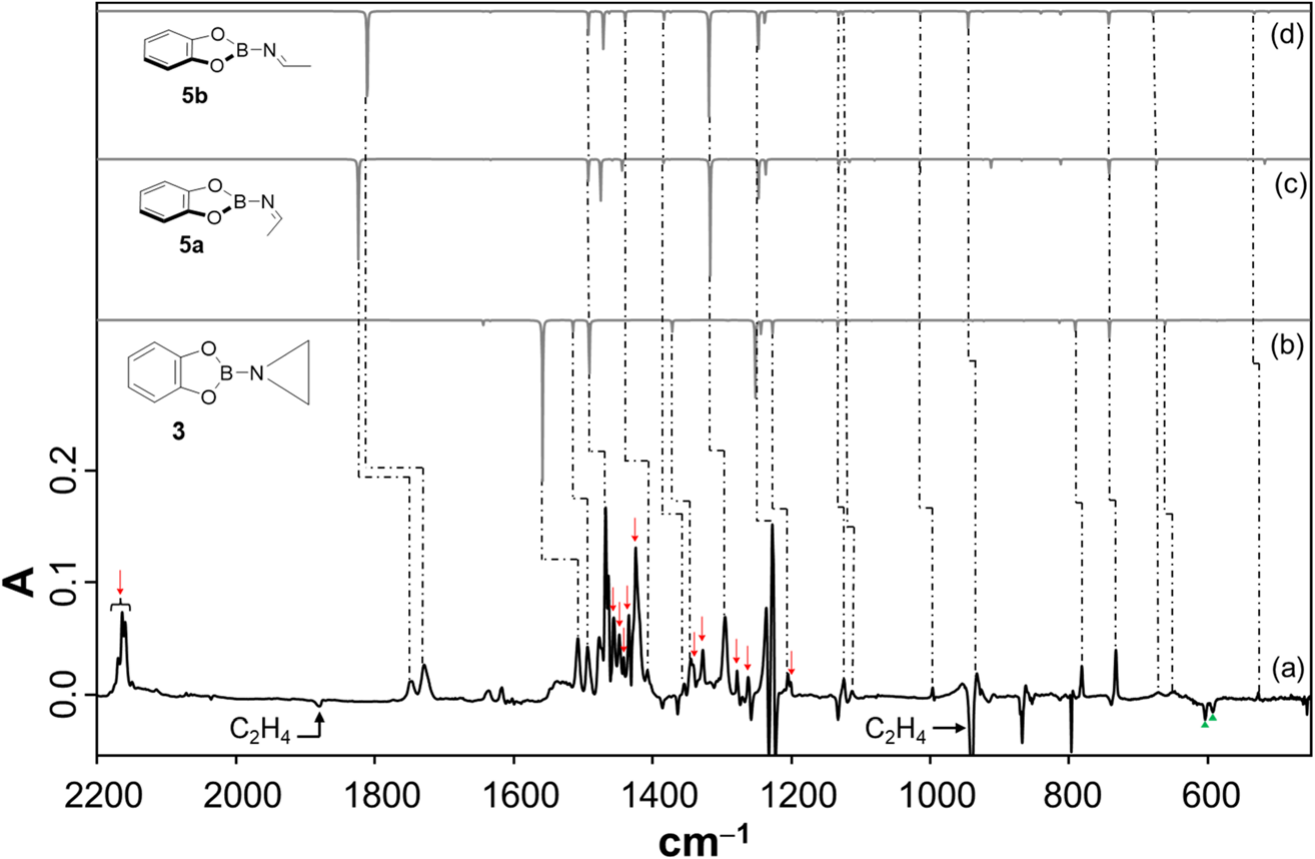
(a) Difference spectrum after irradiating the Ne matrix with *λ* > 550 nm doped with 3% C_2_H_4_ for 120 min; (b–d) harmonic vibrational spectrum (unscaled) for the ^11^B isotopologues of 3, 5a, and 5b, respectively, calculated at the B3LYP/6-311+G(d,p) level of theory; (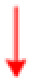) corresponds to the IR bands of 2, 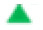 corresponds to the IR bands (see Fig. S2) of ^3^A_2_-1 that show a red shift upon complexation to ethene. Prior to this irradiation, the azide was decomposed to nitrene 1 and N_2_ using *λ* = 254 nm.

**Chart 1:**
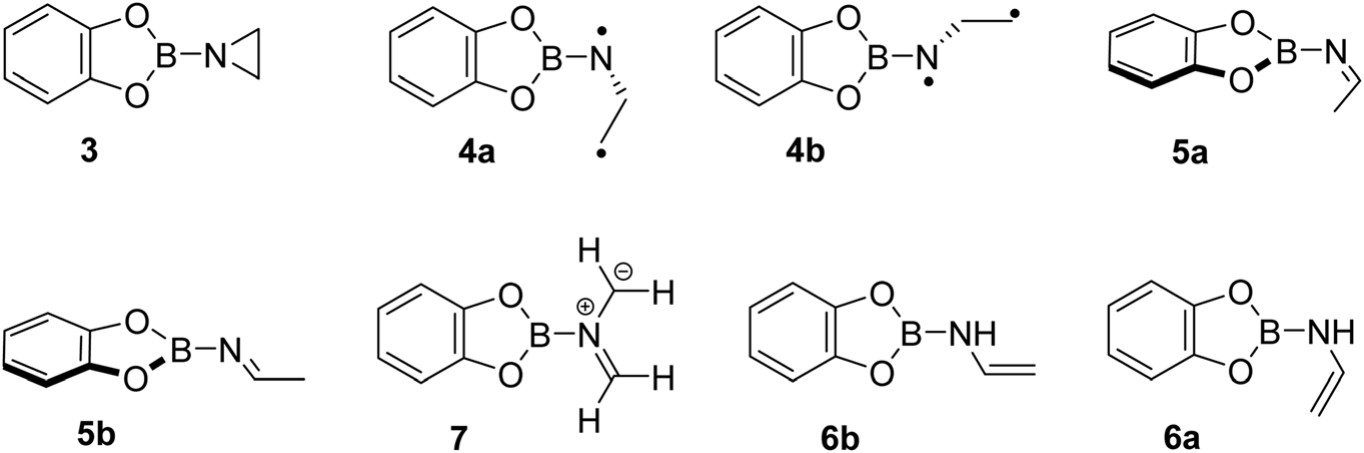
Potential products of the photoreaction between borylnitrene ^3^A_2_-1 and ethene.

To study the reaction of borylnitrene 1 with ethene C_2_H_4_, the borylazide CatBN_3_2 was deposited with a large excess of neon gas doped with 3% C_2_H_4_ onto a cold CsI window maintained at 4 K. Thereafter, the deposited matrix was irradiated with light of wavelength *λ* = 254 nm, which resulted in the formation of borylnitrene 1 in its triplet ground state ^3^A_2_ (Fig. S1a). The infrared (IR) signals assigned to 1 showed a shift compared to the reported IR data,^16^ which is probably due to the complexation^26^ to the dopant C_2_H_4_. In fact, we have experimental evidence supporting the observation of a complex between 1 and ethene (Fig. S2). Especially, the B–N/B–O wagging mode of the complex between 1 and ethene (^3^A_2_-1·C_2_H_4_) show a prominent red shift of 17 cm^−1^ compared to bare ^3^A_2_-1, which matches exactly with the corresponding computed shift. In addition to the signals due to ^3^A_2_-1, weak signals at 1760, 1742, 1520, 1506, and 1307 cm^−1^ were also observed. Subsequently, the matrix was irradiated with *λ* > 550 nm, as performed in our previous studies,^16,19,20,23^ to populate the ^1^A_1_ state of 1 that undergoes intermolecular reaction with C_2_H_4_.^18^ The resulting difference spectrum illustrated in Fig. 1a shows the formation of azide 2 that we observed previously under these conditions,^16,19–23^ the bleaching of IR bands related to C_2_H_4_, and the appearance of many additional IR bands at 1760, 1742, 1520, 1506, 1480, 1475, 1462, 1456, 1419, 1366, 1357, 1307, 1240, 1217, 1137, 1124, 1008, 944, 794, 744, 684, 660, and 539 cm^−1^. The number of new IR bands indicates the formation of more than one species from the photoinduced reaction between ^1^A_1_-1 and ethene. There are a number of potential reaction products of this reaction, including aziridine 3, diastereoisomeric imines 5a and 5b, conformational isomeric enamines 6a and 6b, and azomethine ylide 7 (Chart 1).^27–29^

Based on the computed spectra of these species (Fig. S3), the formation of isomeric enamines 6a and 6b, as well as azomethine ylide 7, can safely be excluded. On the other hand, we surmised the formation of aziridine 3 and imine isomers, based on the IR bands positioned between 1700 and 1800 cm^−1^. To further support the formation of these products, a comparison was made between the experimental difference spectrum resulting from *λ* > 550 nm irradiation and the computed IR spectra of 3, 5a, and 5b, as shown in Fig. 1. The IR bands, such as 1520 and 1217 cm^−1^, are composed of three-membered ring breathing modes and the one at 1506 cm^−1^ is composed of 3-membered ring CH_2_ scissor motion, which are the signature IR bands of three-membered ring compounds.^30^ Additionally, the band at 794 cm^−1^ is predominantly described by the characteristic C–C stretch of the terminal three-membered ring. In the 1800–1700 cm^−1^ region, strong IR bands signify the N=C stretching, while bands at 1307 cm^−1^ and 1366 cm^−1^ are due to the B–N=C–H scissor mode and symmetric bend of the terminal CH_3_ groups, respectively, of 5a and 5b. These IR features are indicative of compounds containing the imine functional group.^31^

**Fig. 2:**
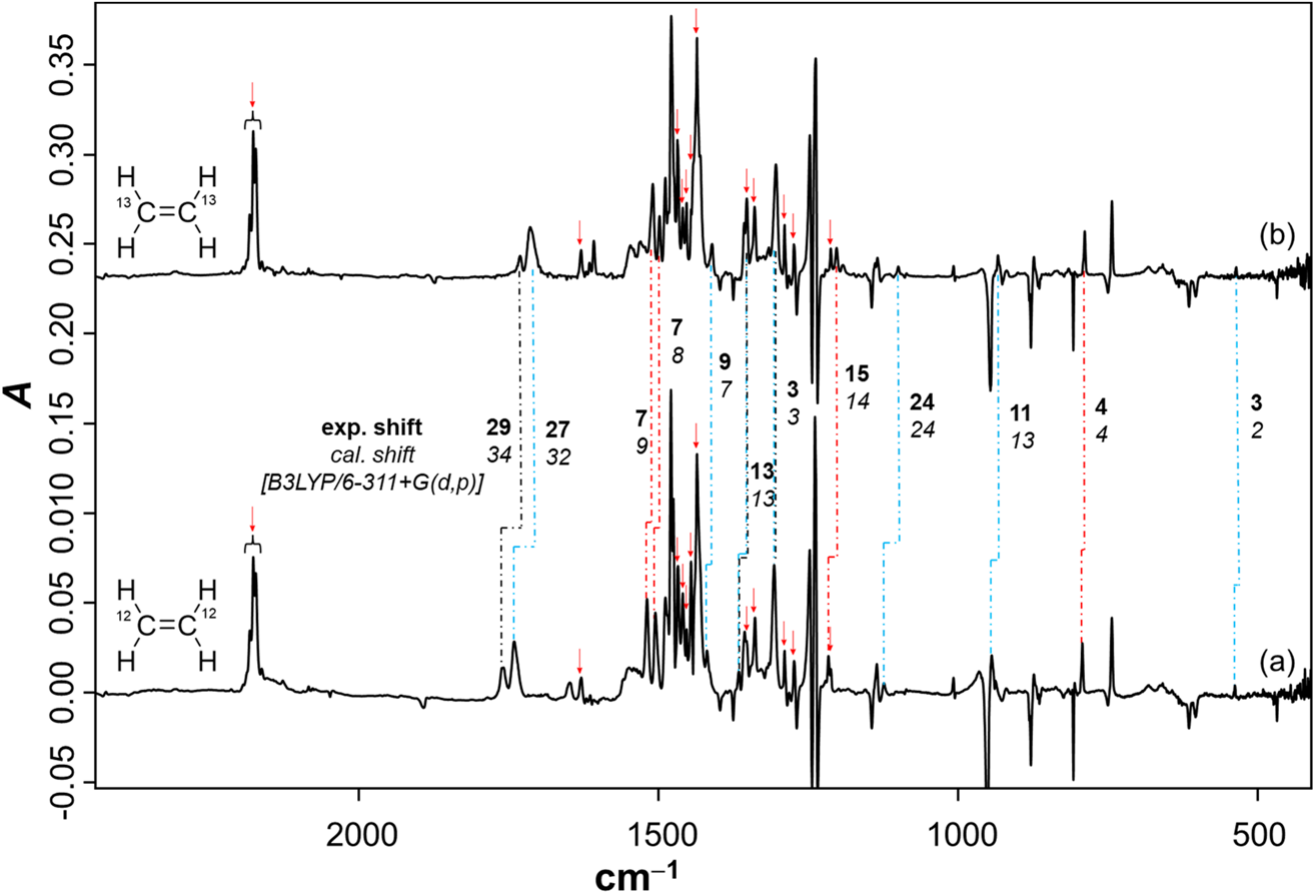
(a) Difference spectrum after irradiating the Ne matrix with *λ* > 550 nm doped with 3% C_2_H_4_ for 120 min; (b) difference spectrum after irradiating the Ne matrix with *λ* > 550 nm doped with 3% ^13^C_2_H_4_ for 120 min; (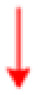) corresponds to the IR bands of 2. (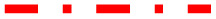), (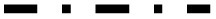), and (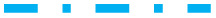) correspond to the experimental isotopic IR band shifts of 3, 5a, and 5b. Prior to this irradiation, the azide was decomposed to nitrene 1 and N_2_ using *λ* = 254 nm.

To further support the spectral assignments, the reaction between 1 and the ^13^C_2_H_4_ isotopologue of ethene was studied. The difference spectra resulting from *λ* > 550 nm irradiation (Fig. 2) show that the characteristic ring breathing modes are red shifted by 7 and 15 cm^−1^, respectively. The CH_2_ scissor mode and the characteristic C–C stretching mode of the terminal three-membered ring are red shifted by 7 cm^−1^ and 4 cm^−1^, respectively. The experimental isotopic shifts match well with the corresponding computed isotopic shifts (Fig. 2). Similarly, the N=C stretches in 5a and 5b show isotopic red shifts of 29 and 27 cm^−1^, which match well with the corresponding computed shifts of 34 and 32 cm^−1^, respectively. Also, the common IR modes in the isomeric imines, CH_3_ symmetric bend and B–N=C–H scissor, show isotopic red shifts of 13 and 3 cm^−1^, respectively, which have a perfect match with the computed shifts. Moreover, the assignment of the products was further corroborated by performing the reaction of ^3^A_2_-1 with C_2_D_4_ (Fig. S4). In addition, the species from the previous step was tested under photoirradiation with *λ* = 254 nm, showing that aziridine 3 remains largely intact while 5b photoisomerizes to 5a (Fig. S10).

**Fig. 3:**
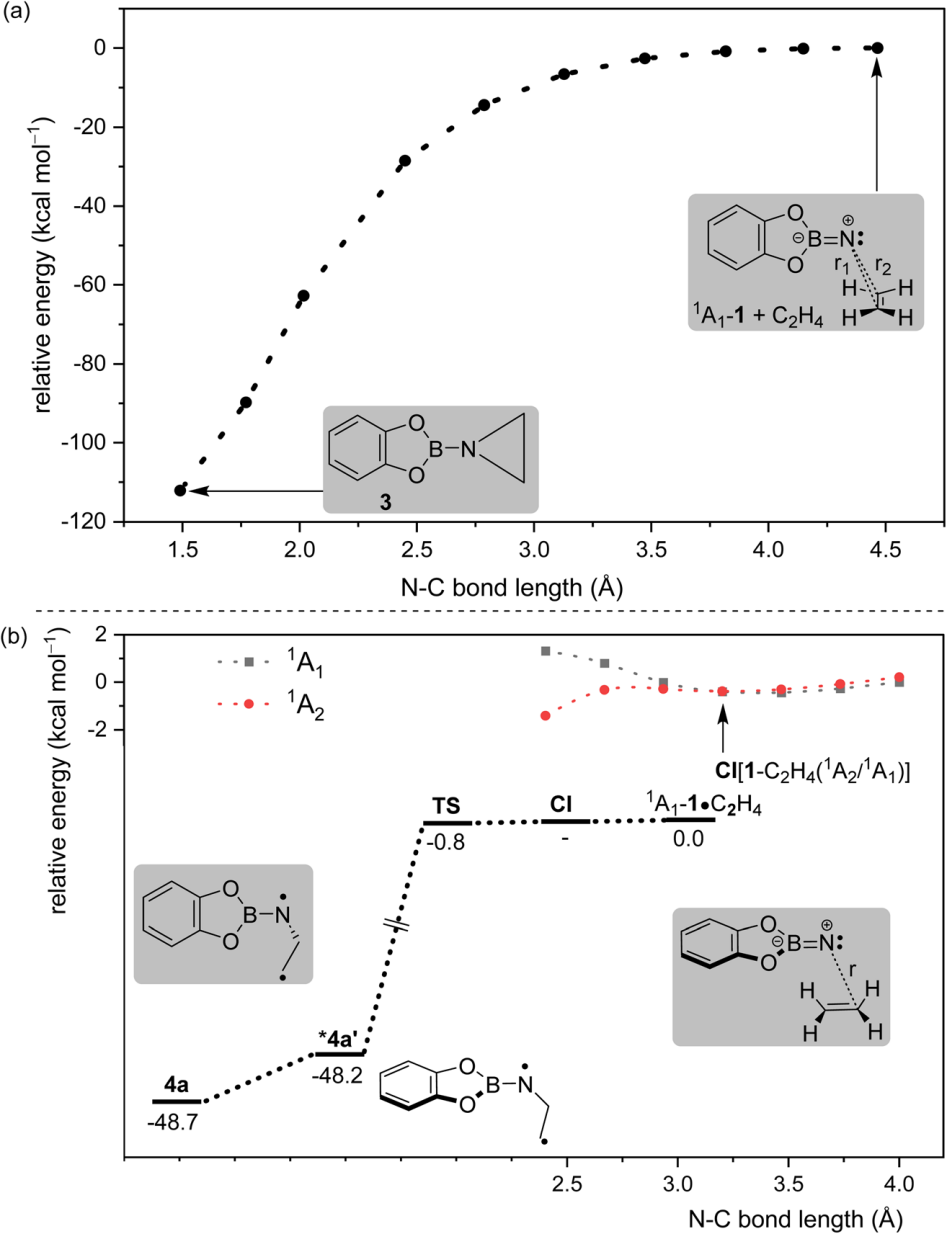
(a) Relaxed potential energy surface scan with constrained bond distances, *r*_1_ and *r*_2_ (*r*_1_ = *r*_2_), between the nitrogen atom of ^1^A_1_-1 and both carbon atoms of ethene calculated at the B2PLYPD3/def2-TZVPP level of theory; (b) lower half: potential energy surface leading to the formation of *syn*-1,3-diradical 4a starting from the complex ^1^A_1_-1·C_2_H_4_ calculated at the CASPT2/def2-TZVPP//CASPT2/def2-SV(P) level of theory; upper right corner: partial relaxed potential energy surface scan in a bisected manner (similar to ^1^A_1_-1·C_2_H_4_) with a constrained bond distance *r* between the nitrogen atom of 1 and one of the carbon atoms of ethene, calculated at the CASPT2/def2-TZVPP//B2PLYPD3/def2-TZVPP level of theory (see the SI for details of the CASPT2 calculations). The conical intersection (CI) is marked by an arrow. * Second-order saddle point.

The unexpected formation of isomeric imines necessitated further computational investigation to understand the reaction mechanism. Previously, we have obtained computational evidence that the irradiation of 1 in its triplet ground state with *λ* > 550 nm populates the ^1^A_1_ state.^18^ Therefore, we consider the ^1^A_1_ state as the starting point of our computational investigations that comprise two components: (1) the formation of primary products resulting from the reaction between ^1^A_1_-1 and ethene, and (2) the subsequent formation of secondary products. First, we studied the formation of the primary product aziridine 3*via* a relaxed potential energy surface (PES) scan. The scan parameters *r*_1_ and *r*_2_, as shown in Fig. 3a, were constrained to the same value at each step of the scan to ensure *quasi-C*_s_ symmetry throughout the process. The PES has no barrier for the formation of aziridine and therefore supports the experimental observation of 3.

In addition, the reaction was studied with a different reaction coordinate, as shown in Fig. 3b, as the second part of the primary reaction. In this reaction, the planes containing 1 and ethene lie parallel to each other at the start of the reaction (pre-reactive complex). The pre-reactive complex is a good starting point considering the available experimental evidence for such a complex, as discussed above (Fig. S2). Considering the possibility of the multireference character of the wavefunctions of different species along this reaction coordinate, complete active space second-order perturbation (CASPT2) theory was chosen. The calculated stationary points show the barrierless formation of 1,3-diradical 4a (Fig. 3b). A partial PES scan was performed with *r* as the scan parameter (Fig. 3b) to show the transfer of population from initially populated ^1^A_1_ to the ^1^A_2_ state of 1 along such a reaction coordinate through a conical intersection (CI).

**Fig. 4:**
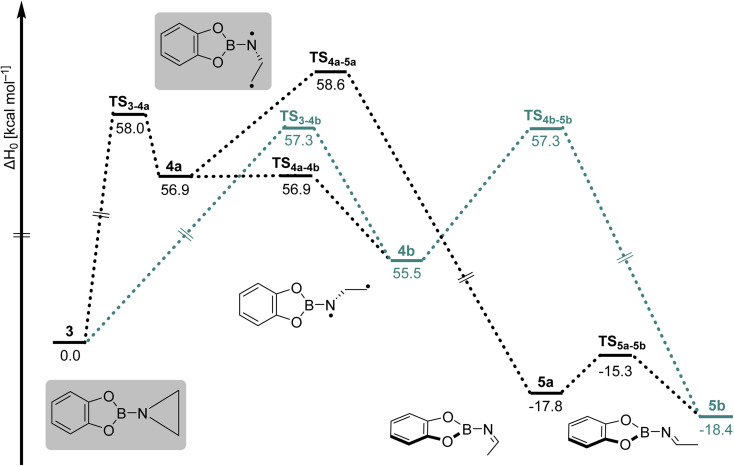
Potential energy surface initiating from aziridine 3 and *syn*-1,3-diradical 4a calculated at the B2PLYPD3/def2-TZVPP level of theory.

The observation of imine products in the experimental studies could be explained by the second part of our computational studies. Herein, we have computed a PES starting from aziridine 3 and 1,3-diradical 4a, as shown in Fig. 4 (highlighted in grey). Conversion of 3 into isomeric imines 5a and 5b can proceed *via* isomeric 1,3-diradicals 4a and 4b, respectively. Since these steps involve high energy barriers of around 57–59 kcal mol^−1^, these paths are not feasible for the formation of isomeric imines under the cryogenic conditions employed in our experiments if energy transfer to the surrounding matrix is efficient. Furthermore, IR measurements of the ^13^C-labeled reaction (Fig. S11) at various time intervals reveal the formation of 5a/5b without accumulation of 3, suggesting that the imines form independently rather than as secondary photoproducts. On the other hand, the formation of 5a*via* a 1,2-H shift to the terminal carbon atom of 4a has a barrier of merely 1.7 kcal mol^−1^, which can be surpassed in cryogenic matrices at 4 K. In addition, the conversion of 4a into 4b has a negligible barrier, which in turn has an accessible energy barrier of 1.8 kcal mol^−1^ for its conversion into 5b. Furthermore, starting from 4a and 4b, we explored the possibility of forming enamine products 6a and 6b through a 1,2-H shift to the nitrogen atom, as shown in Fig. S5. High barriers for the formation of isomeric enamines from 4a and 4b, compared to the isomeric imines, support their experimental absence.

## Conclusions

**Scheme 3:**
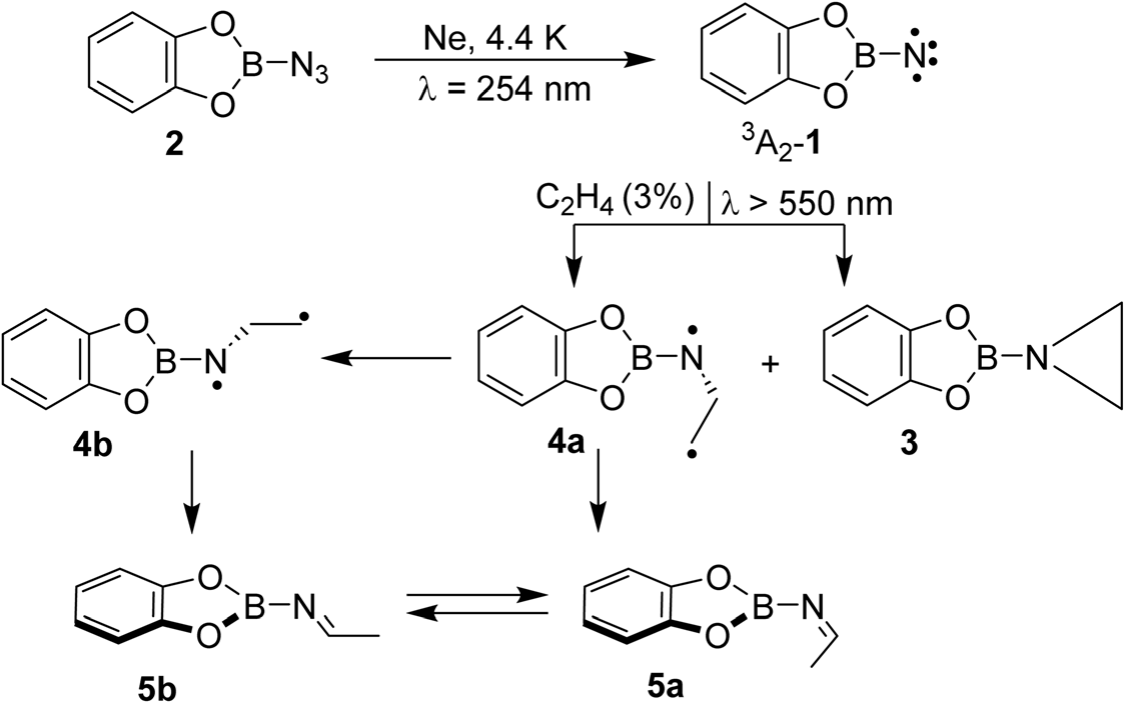
Formation of aziridine 3 and isomeric imines 5a and 5b resulting from the reaction of borylnitrene 1 and ethene.

The combined matrix isolation and computational studies regarding the photochemical reactivity of 1 with ethene demonstrate the formation of aziridine 3 and isomeric imines 5a and 5b, as shown in Scheme 3. Notably, the formation of isomeric imines is rare for such a reaction. Computational studies support the formation of singlet 1,3-diradical 4a, which could not be observed experimentally, as an alternate primary product alongside the expected aziridine 3. Finally, the diradical 4a has accessible energy barriers for its conversion into imines 5a and 5b. The observations are remarkable as the formation of the 1,3-diradical intermediate in cycloaddition reactions of nitrenes is the hallmark of triplet state reactivity, while here the singlet state is involved. The low barrier for collapse of the diradical to the imines 5 and aziridine 3 suggests that these processes are very fast. We are currently investigating the dual mode reactions of borylnitrenes with olefins in solution to shed more light onto this unusual reactivity.

## Author contributions

V. B. conducted all calculations, experiments involving matrix isolation, and data analysis. V. B. and H. F. B. jointly wrote the first draft of the manuscript. H. F. B. conceived the research idea, revised the manuscript, and secured funding.

## Conflicts of interest

The authors declare no competing interests.

## Supplementary Material

SC-OLF-D5SC07994B-s001

## Data Availability

The data that support the findings of this study are available in the supplementary information (SI) of this article. Supplementary information: matrix isolation spectra, peak tables, computational details, and Cartesian coordinates. See DOI: https://doi.org/10.1039/d5sc07994b.
