# Supplementary material for: Unusual nitrene reactivity: imine formation in the photochemical reaction of a borylnitrene with ethene
Source: Chem Sci. 2025 Dec 24;17(7):3651–7. doi: 10.1039/d5sc07994b (PMC12728564; doi:10.1039/d5sc07994b)
Supplement: SC-017-D5SC07994B-s001 [file SC-017-D5SC07994B-s001.pdf]

Supplementary Information (SI) for Chemical Science.
This journal is © The Royal Society of Chemistry 2025

Supporting Information

Unusual Nitrene Reactivity: Imine Formation in the Photochemical Reaction of Borylnitrene with Ethene

Virinder Bhagat, Holger F. Bettinger*

Institut für Organische Chemie, Universität Tübingen, Auf der Morgenstelle 18, 72076 Tübingen, Germany

holger.bettinger@uni-tuebingen.de

Materials and Methods

Matrix Isolation Experiments:

The precursor, **2**, of the borylnitrene **1** was synthesized using the procedure as followed by Klapötke et al.[1] For the matrix host, gaseous neon (Messer-Griesheim, 99.9999%) was used, which was doped with 3% of different isologues of ethene (C_2H_4, Messer, 99.95%; C_2D_4, Aldrich, 99% D; $^{13}C_2H_4$, Sigma-Aldrich, 99% ^{13}C) in separate experiments. For performing matrix isolation experiments, a SHI CKW-21A displex closed-cycle helium cryostat was used. Additionally, Edwards' oil diffusion pump was used to maintain a high vacuum of 10^{-6} mbar during the experiments. During deposition, the precursor was immersed in a cold bath maintained at 0° Celsius, and it was co-deposited with a huge excess of doped neon using an MKS PR4000B mass-flow controller at a flow rate of 2 sccm. For photochemistry at different steps, a high-pressure mercury lamp (USHIO, USH-508S) and a low-pressure mercury lamp ($\lambda = 254$ nm, PenRay) were used. The experimental data were analyzed using IR spectroscopy with a Bruker Vertex 70 spectrophotometer with a standard resolution of 0.5 cm^{-1}

Computational Details:

For comparison with the experimental IR band, the IR spectra of different species were computed using B3LYP[2, 3] functional in conjunction with 6-311+G(d,p) basis set.[4, 5] For the reaction mechanism studies, geometry optimization was performed using the B2PLYPD3 functional.[6, 7] For species prone to multireference character (Fig. 3b), rs2c formalism of complete active space perturbation theory (CASPT2)[8, 9] was employed using default parameters. For the active spaces used for different species, please refer to Fig. S8 and Fig. S9. The Ahlrichs' def2-TZVPP and def2-SV(P) basis set[10] was used in combination with B2PLYPD3 and CASPT2. For B2PLYPD3, geometry optimization and frequency analysis were conducted analytically, while similar calculations were performed numerically for CASPT2 theory. All the DFT computations were carried out using the Gaussian16 program package[11] and the CASPT2 calculations were carried out using Molpro v. 2024.1.[12]

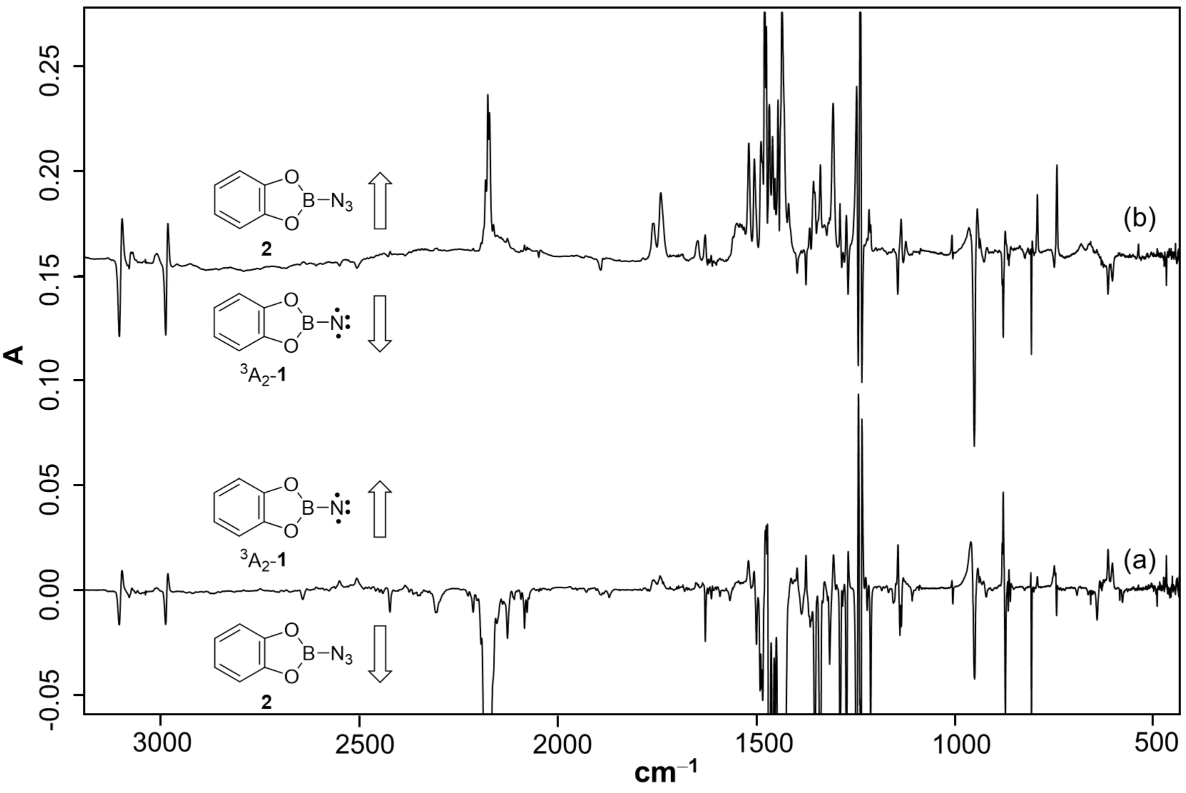

Fig. S1. (a) Difference spectrum after irradiating the Ne matrix with λ > 254 nm doped with 3% of C_2H_4 for 10 min. (b) Difference spectrum after irradiating the Ne matrix with λ > 550 nm doped with 3% of C_2H_4 for 120 min.

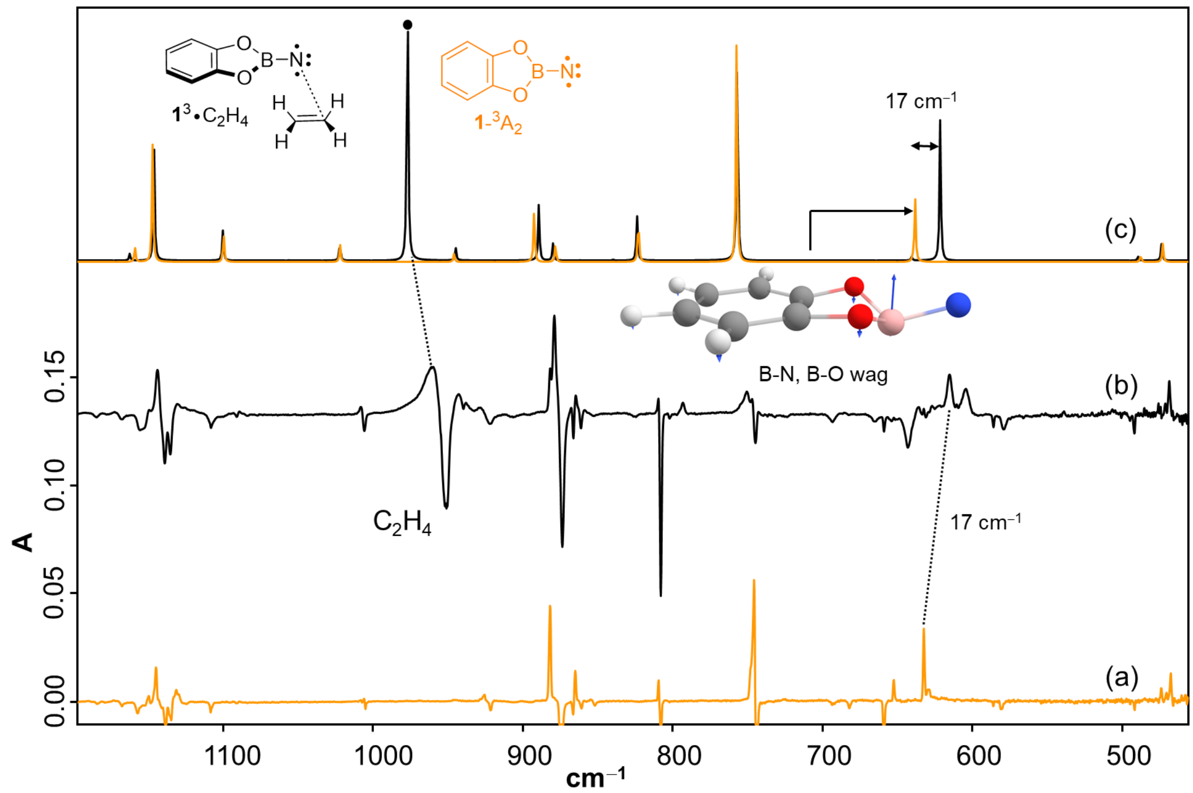

Fig. S2. (a) Difference spectrum after irradiating the Ne matrix containing **2** with $\lambda > 254$ nm for 10 min. (b) Difference spectrum after irradiating the Ne matrix doped with 3% of C_2H_4 and containing **2** with $\lambda > 254$ nm for 10 min. (c) Calculated harmonic spectra (unscaled; fundamental bands) for ^{11}B isotopologues of $^3A_2$-**1** and $^3A_2$-**1** complexed to ethene (1^3•C_2H_4) calculated at (U)B3LYP-D3/6-311+g(d,p). ● Correspond to the IR bands of the terminal C_2H_4 unit in 1^3•C_2H_4.

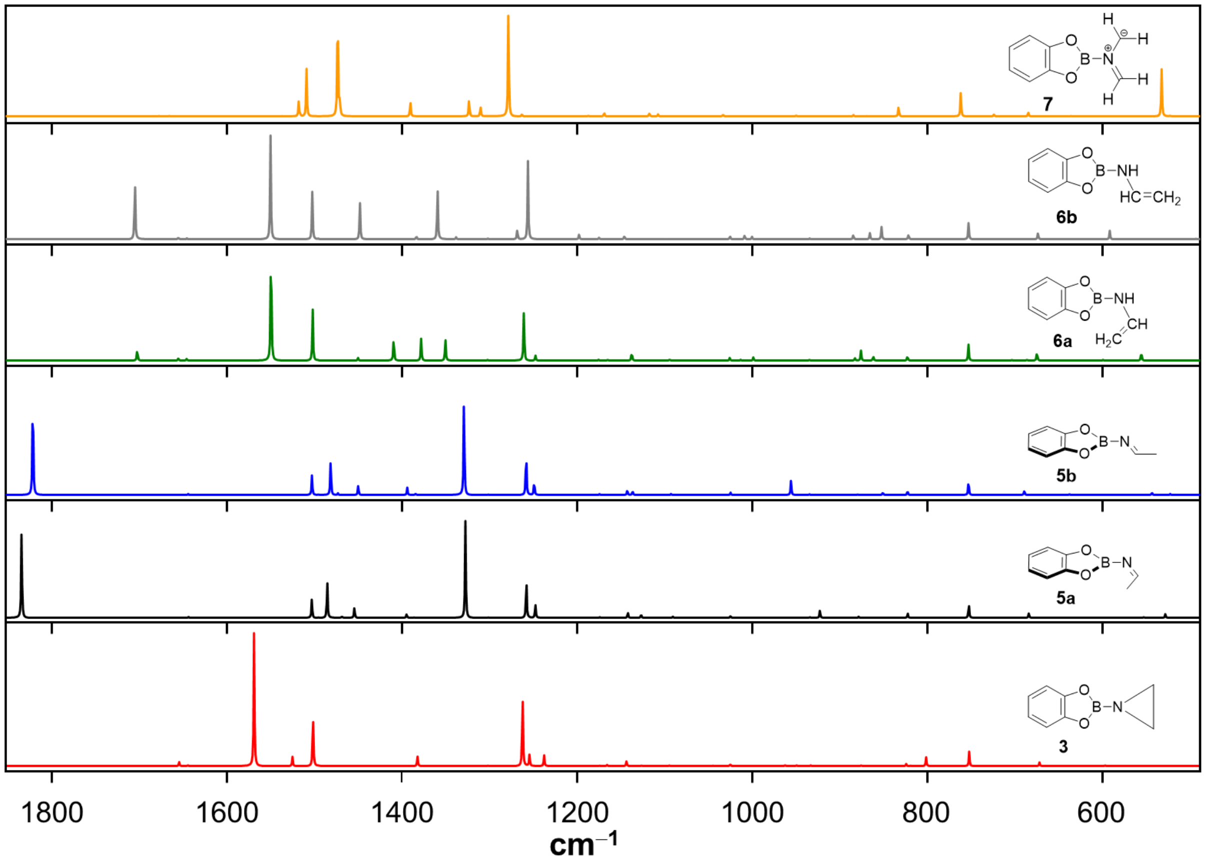

Fig. S3. Calculated harmonic spectra (unscaled; fundamental bands) for ^{11}B isotopologues of the probable products of the photochemical reaction of borylnitrene **1** and ethene at B3LYP/6311+G(d,p) level of theory.

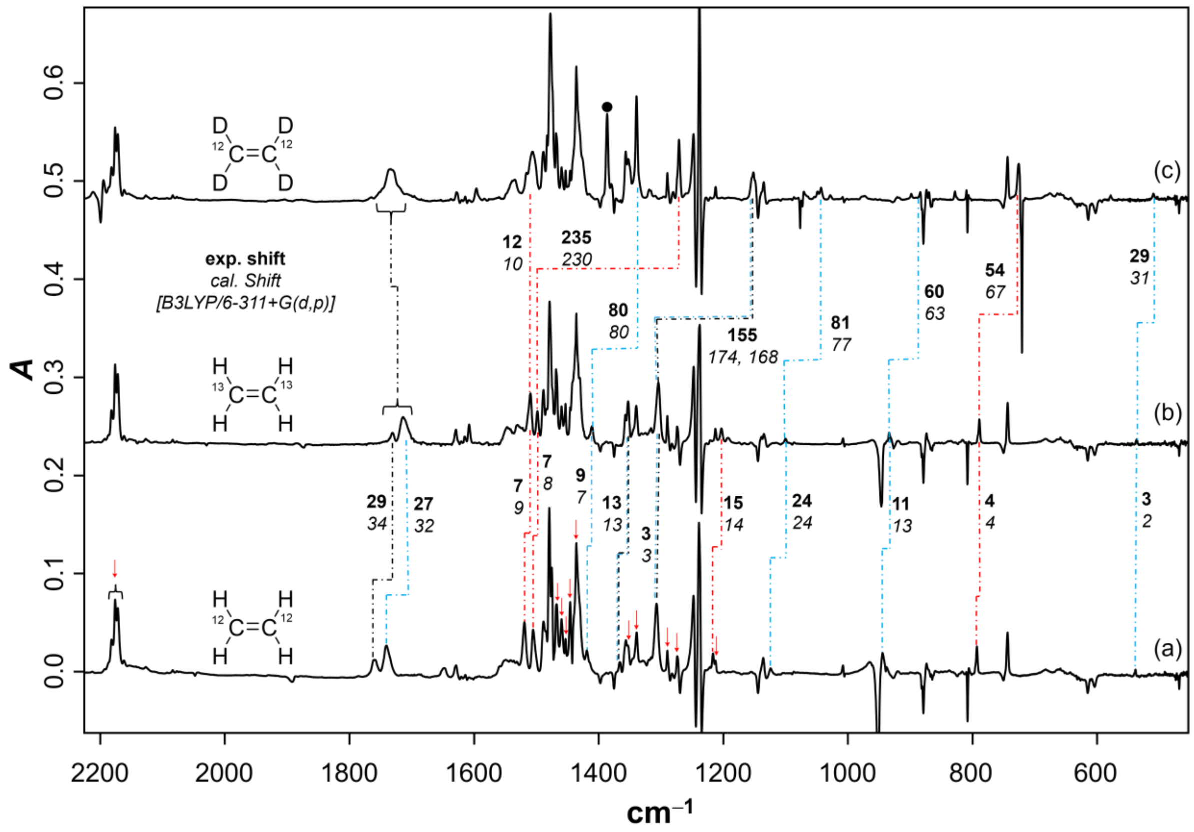

Fig. S4. Difference spectrum after irradiating Ne matrix with λ > 550 nm doped with (a) 3% of C_2H_4, (b) 3% of $^{13}C_2H_4$, and (c) 3% of C_2D_4 for 120 min. (↓) correspond to the IR bands of **2**. (-·-·-), (-·-·-), and (-·-·-) correspond to the experimental isotopic IR band shifts of **3**, **5a**, and **5b**. ● Similar band in spectrum (a) overlaps with the strong bands of precussor **2**. Prior to this irradiation, the azide was decomposed to nitrene **1** and N_2 using λ = 254 nm.

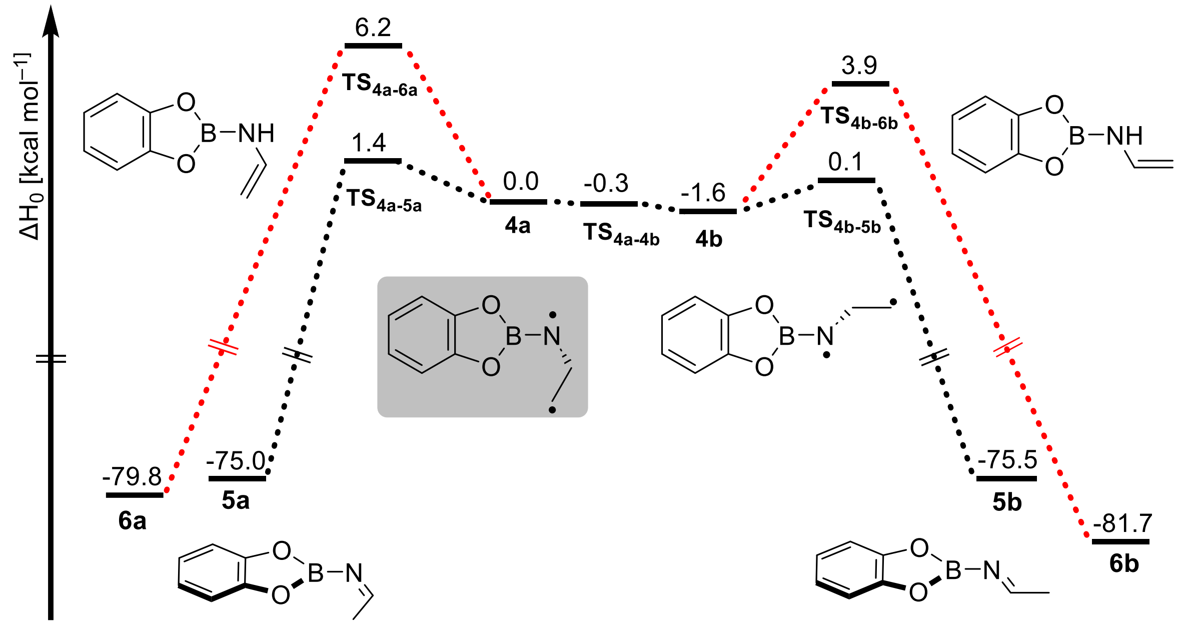

Fig. S5. Potential energy surface connecting *syn*-1,3-diradical **4a** with *syn* (**5a** and **6a**) and *anti* products (**5b** and **6b**), calculated at the B2PLYPD3/def2-TZVPP//B2PLYPD3/def2-SV(P) level of theory.

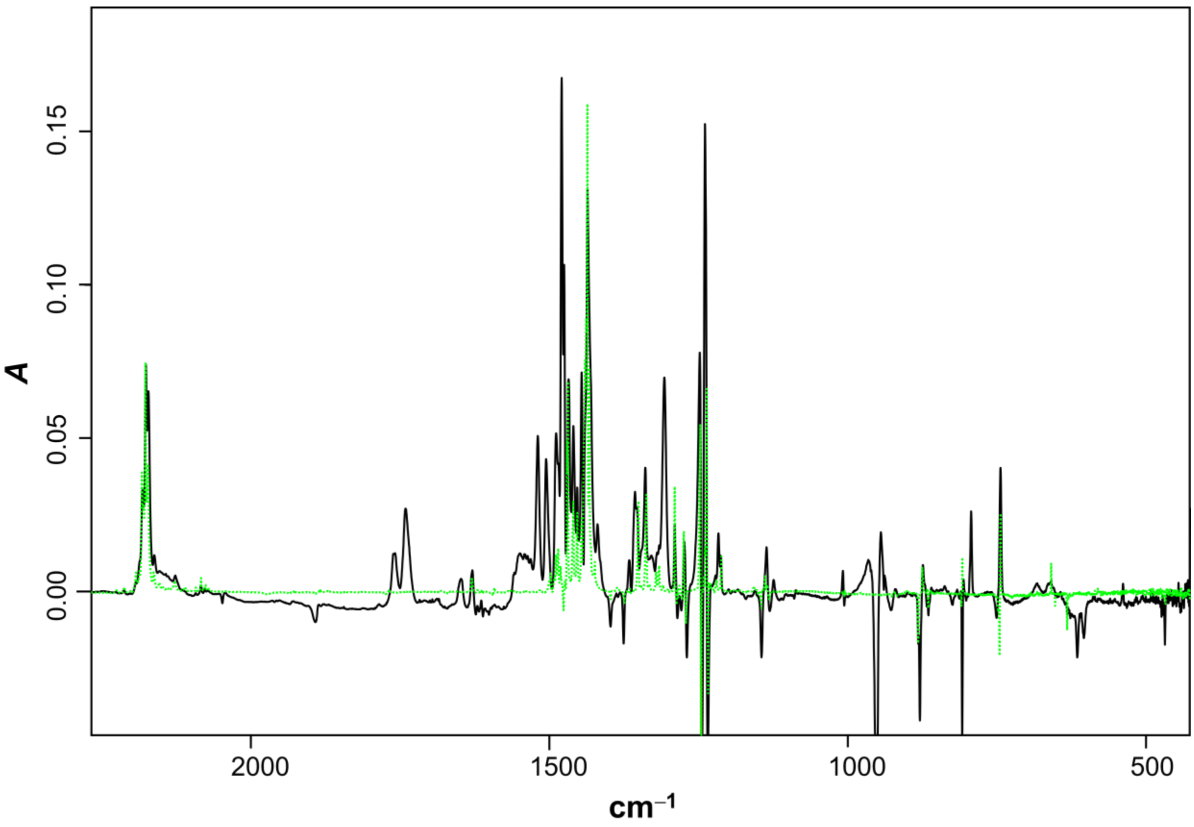

Fig. S6. Difference spectrum after irradiating ($\lambda > 550$ nm) the Ne matrix with (black and solid) and without (green and dotted) doping with 3% of C_2H_4 for 120 min and 45 min, respectively.

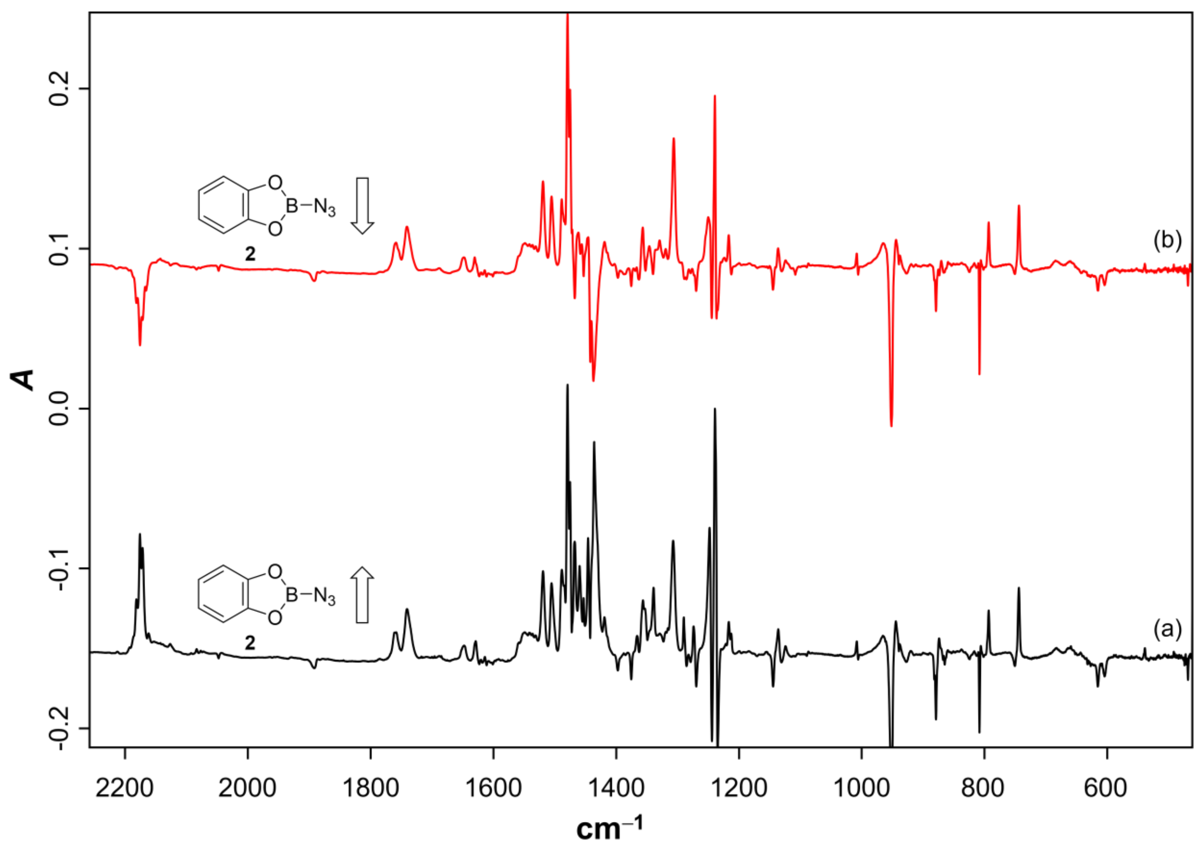

Fig. S7. (a) Difference spectrum after irradiating (λ > 550 nm) the Ne matrix doped with 3% of C_2H_4 for 120 min; b) Difference spectrum after irradiating the Ne matrix with λ = 254 nm (following λ > 550 nm) for 8 min. Prior to λ > 550 nm irradiation, the azide was decomposed to nitrene **1** and N_2 using λ = 254 nm.

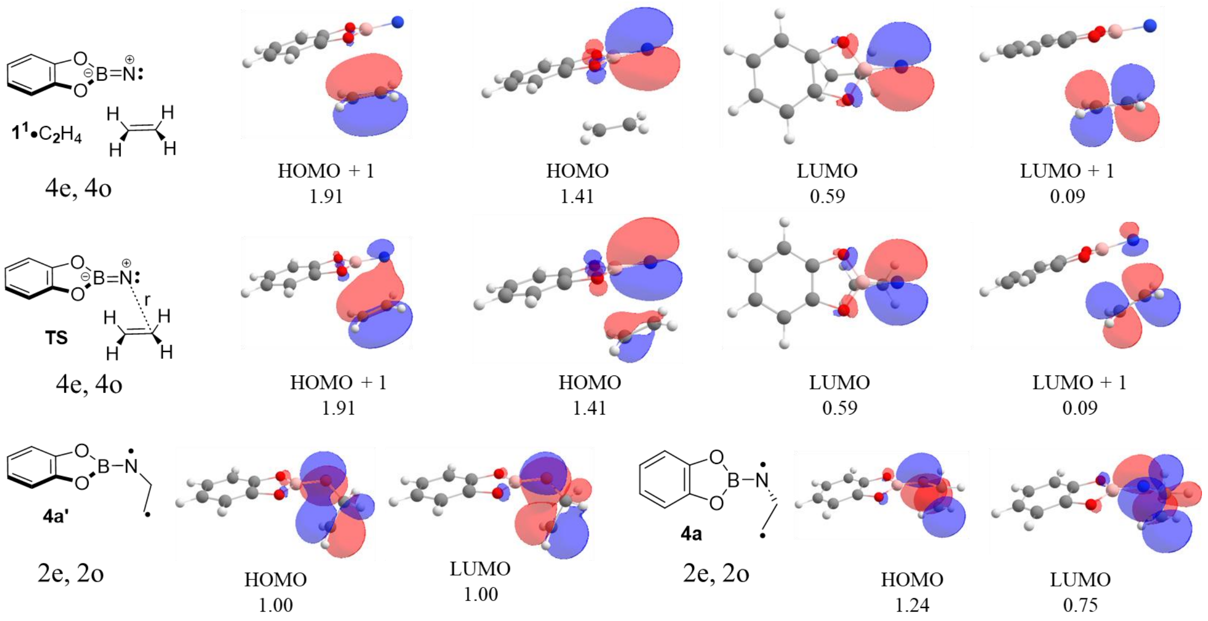

Fig. S8. Active space (x electrons, y orbitals) orbitals and their occupation number obtained in CASPT2/def2-SV(P) geometry optimization.

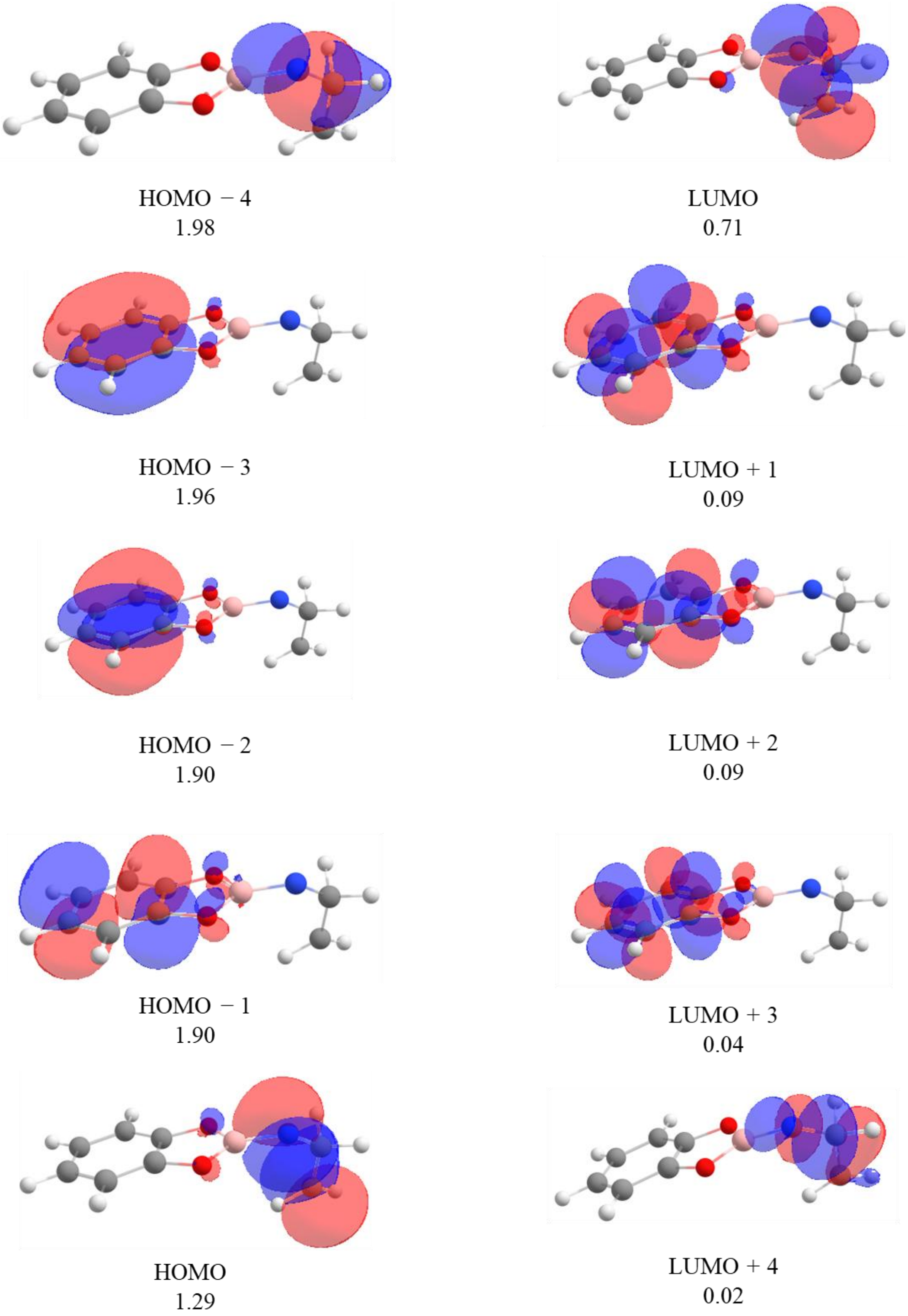

Fig. S9. Active space (10 electrons, 10 orbitals) orbitals and their occupation number obtained in CASPT2/def2-TZVPP//CASPT2/def2-SV(P) calculations.

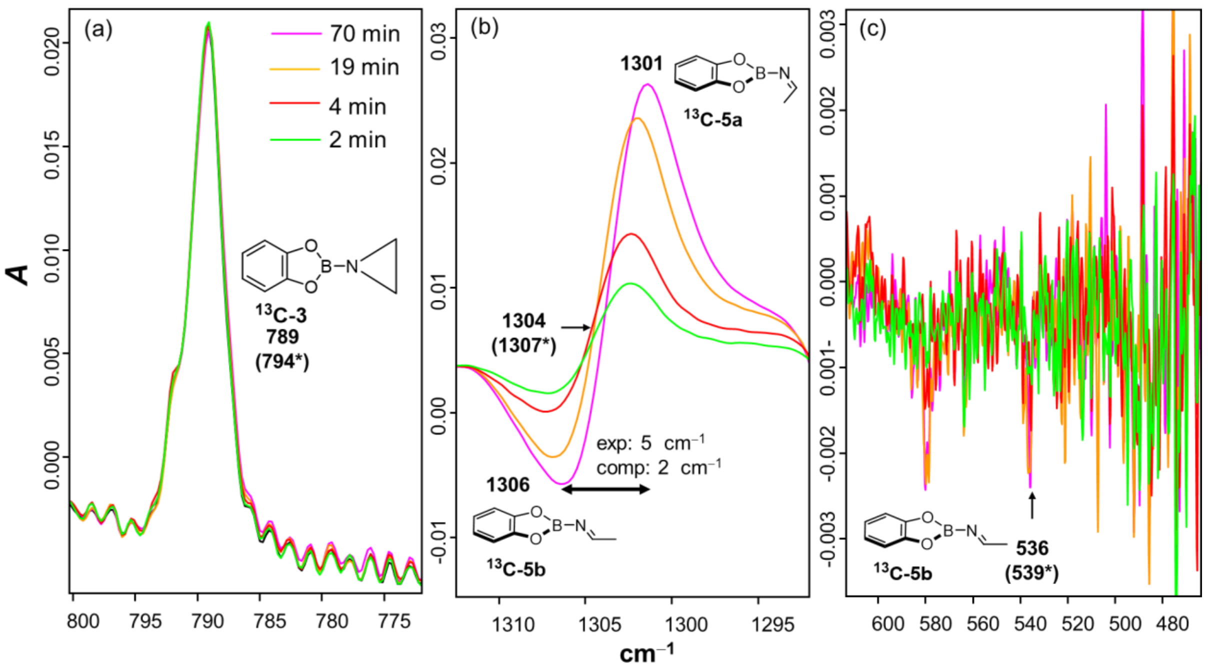

Fig. S10. (a) Effect of photoirradiating the matrix with λ = 254 nm following the λ > 550 nm photoirradiation step to study the photochemistry of ^{13}C isotopologues of **3**, **5a**, and **5b**. * The corresponding IR bands of unlabeled **3**, **5a**, and **5b**. * The corresponding IR bands of unlabeled **3**, **5a**, and **5b**.

The above results show the outcome of the photochemistry of ^{13}C isotopologues of **3**, **5a**, and **5b** upon λ = 254 nm photoirradiation. To study the photochemistry, we have chosen the IR bands of ^{13}C isotopologues **3** (789 cm^{-1}), **5a** (1304 cm^{-1}), and **5b** (1304 cm^{-1} and 536 cm^{-1}), which are intense and isolated in the IR spectra and therefore easy to analyze. According to Fig. S10(a), aziridine **3** mostly remains intact upon photoirradiation with λ = 254 nm over different periods of time. Additionally, based on Fig. S10(b) and Fig. S10(c), **^{13}C-5b** undergoes photoisomerization to **^{13}C-5a**. The band at 1304 cm^{-1}, which appears as a common band for **^{13}C-5a** and **^{13}C-5b** due to the minimal difference between their corresponding bands, can be observed as two separate bands at 1306 cm^{-1} and 1301 cm^{-1}.

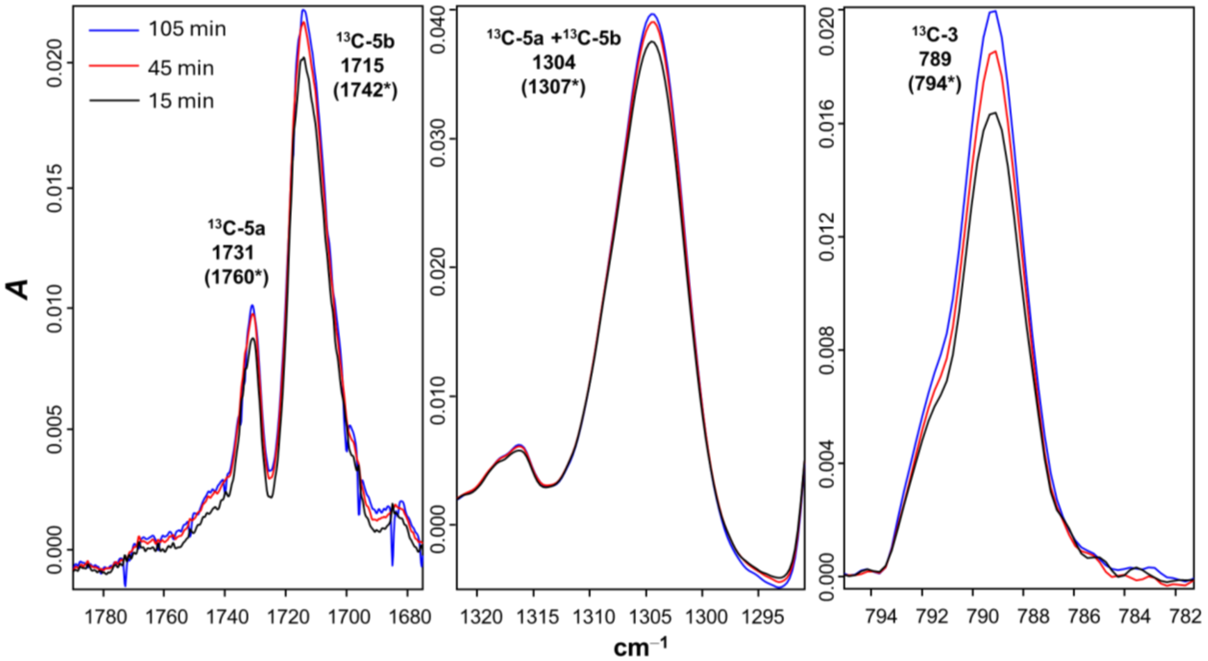

Fig. S11. (a) Intense IR bands correspond to ^{13}C isotopologue of **3**, **5a**, and **5b** obtained from the difference IR spectrum after irradiating (λ > 550 nm) the Ne matrix doped with 3% of C_2H_4 for different intervals of time. * The corresponding IR bands of unlabeled **3**, **5a**, and **5b**.

Table S1. Infrared spectroscopic data of observed and computed (harmonic and unscaled, B3LYP/6-311+G(d,p)) vibrational frequencies of the ^{11}B isotopologue of **3** in the neon matrix.

Vibrational Mode, ν	Experimental	Computed		approx. description
	ν (cm^{-1})[a]	ν (cm^{-1})	I[b]	
1	–	3203	0.3	aromatic C-H str
2	–	3199	0.9	aromatic C-H str
3	–	3186	1.4	aromatic C-H str
4	–	3180	3.5	aziridine C-H str
5	–	3172	0.2	aromatic C-H str
6	–	3167	0	aziridine C-H str
7	–	3092	6.4	aziridine C-H str
8	–	3089	3.1	aziridine C-H str
9	–	1655	3	ring str
10	–	1645	0.6	ring str
11	1520	1569	100[c]	B-N str, aziridine ring breathing
12	1506	1526	6.9	CH_2 scissors
13	1475	1502	41.6	skel. ring, in pl. C-H
14	–	1500	0.1	CH_2 scissors
15	–	1496	0.2	skel. ring, in pl. C-H
16	1357	1383	7.5	skel. ring
17	–	1302	0.3	in plane C-H
18	1240	1263	55	B-N, C-O, ring str
19	–	1255	8.7	asym B-O str
20	1217	1238	8.2	skel. ring, aziridine ring breathing
21	–	1175	0.3	skel. ring, in pl. C-H
22	–	1166	0.9	CH_2 wagging
23	–	1163	0	CH_2 rocking
24	1137	1144	3.6	skel. ring, in pl. str
25	–	1141	0.1	CH_2 twist
26	–	1127	0.5	CH_2 wagging
27	–	1095	0.3	skel. ring, in pl. str
28	–	1045	0.3	CH_2 twist
29	1008	1026	1.4	skel. ring, in pl. str
30	–	974	0	C-H wag
31	–	963	0.7	aziridine ring str
32	–	950	0.5	aziridine ring deformation
33	–	934	0.5	C-H wag
34	–	876	0.5	skel. ring, C-O str
35	–	859	0	C-H wag
36	–	825	1.6	ring breathing, CH2 wag
37	–	820	0.3	CH_2 scissors
38	794	802	6.7	bicylus breathing, terminal C–C stretch
39	744	753	10.8	C-H wag
40	–	743	0	ring out of plane
41	660	673	2.7	B-N, B-O wag

42	–	622	0	in pl. ring deformation
43	–	598	0.5	in pl. ring deformation
44	–	581	0	ring out of plane
45	–	536	0	In pl. ring deformation
46	–	432	0.9	ring out of plane

[a]Obtained after irradiating neon matrix containing **2** and doped with 3 % of C_2H_2 for 120 min; [b]Intensities are relative to the strongest band; [c]Computed absolute intensity: 909.8 km mol^{-1}.

Table S2. Infrared spectroscopic data of observed and computed (harmonic and unscaled, B3LYP/6-311+G(d,p)) vibrational frequencies of the ^{11}B isotopologue of **5a** in the neon matrix.

Vibrational Mode, ν	Experimental	Computed		approx. description
	ν (cm^{-1})[a]	ν (cm^{-1})	I[b]	
1	–	3203	0.4	aromatic C-H str
2	–	3200	1.2	aromatic C-H str
3	–	3186	2	aromatic C-H str
4	–	3172	0.2	aromatic C-H str
5	–	3119	3	alkyl C-H str
6	–	3073	1.1	alkyl C-H str
7	–	3021	4	alkyl C-H str
8	–	3014	8.8	alkyl C-H str
9	1760	1835	93.9	N=C str
10	–	1654	0.3	ring str
11	–	1644	1	ring str
12	1480	1503	20.3	skel. ring, in pl. C-H
13	-	1496	0.3	skel. ring, in pl. C-H
14	1462*	1486	39.3	CH_3 asym. bend, HCN bend
15	–	1469	1.4	CH_3 twist
16	–	1455	11.5	CH_3 asym. bend, HCN bend
17	1366	1395	4.1	CH_3 sym. bend
18	–	1386	0.5	skel. ring
19	1307	1328	100[c]	NCH in pl. bend
20	–	1302	0.5	in plane C-H
21	1240	1259	42.8	B-N, C-O, ring str.
22	–	1248	14.6	asym B-O str.
23	–	1175	0.8	skel. ring, in pl. C-H
24	1137	1143	5.9	skel. ring, in pl. str
25	–	1127	3.8	CNH in pl. bend
26	–	1101	0.1	CH_3 rocking, CNH rocking
27	–	1091	1.2	skel. ring, in pl. str
28	1008	1025	2	skel. ring, in pl. str
29	–	975	0	C-H wag
30	–	935	0.7	C-H wag
31	–	923	7.8	terminal C-C stretch
32	–	879	1.2	skel. ring, C-O str
33	–	860	0	C-H wag
34	–	850	0.2	bicylus breathing
35	–	833	0	CNH rocking
36	–	823	4.8	ring breathing
37	744	753	15.4	C-H wag
38	–	744	0	ring out of plane
39	684	685	5	B-N, B-O wag
40	–	621	0	in pl. ring deformation
41	–	617	0	in pl. ring deformation
42	–	581	0	ring out of plane

43	–	553	0.7	In pl. ring deformation
44	–	529	4.1	BNC bending
45	–	447	0.4	ring stretching
46	–	430	0.7	ring out of plane

[a]Obtained after irradiating neon matrix containing **5a** and doped with 3 % of C_2H_2 for 120 min; [b]Intensities are relative to the strongest band; [c]Computed absolute intensity: 642.4 km mol^{-1}; * could be observed only in Fig. S7b.

Table S3. Infrared spectroscopic data of observed and computed (harmonic and unscaled, B3LYP/6-311+G(d,p)) vibrational frequencies of the ^{11}B isotopologue of **5b** in the neon matrix.

Vibrational Mode, ν	Experimental	Computed		approx. description
	ν (cm^{-1})[a]	ν (cm^{-1})	I[b]	
1	–	3203	0.4	aromatic C-H str
2	–	3200	1.1	aromatic C-H str
3	–	3186	1.8	aromatic C-H str
4	–	3172	0.2	aromatic C-H str
5	–	3132	1.4	alkyl C-H str
6	–	3069	1.3	alkyl C-H str
7	–	3020	1.4	alkyl C-H str
8	–	2963	10.3	alkyl C-H str
9	1742	1822	100[c]	N=C str
10	–	1654	0.2	ring str
11	–	1645	0.9	ring str
12	1480	1503	18.6	skel. ring, in pl. C-H
13	–	1496	0.3	skel. ring, in pl. C-H
14	1456*	1482	32.6	CH_3 asym. bend, HCN bend
15	–	1474	1.6	CH_3 twist
16	1419	1450	9.2	CH_3 asym. bend, HCN bend
17	1366	1394	6.6	CH_3 sym. bend
18	–	1385	1.2	skel. ring
19	1307	1330	91.9	NCH in pl. bend
20	–	1302	0.4	in plane C-H
21	1240	1259	41.6	B-N, C-O, ring str.
22	–	1249	12.8	asym B-O str.
23	–	1175	0.8	skel. ring, in pl. C-H
24	1137	1143	4.9	skel. ring, in pl. str
25	1124	1137	4	CNH in pl. bend
26	–	1105	0.1	CH_3 rocking, CNH rocking
27	–	1093	0.9	skel. ring, in pl. str
28	1008	1025	2.1	skel. ring, in pl. str
29	–	975	0	C-H wag
30	944	956	13.4	terminal C-C stretch
31	–	935	0.7	C-H wag
32	–	880	0.4	skel. ring, C-O str
33	–	860	0	C-H wag
34	–	852	0.6	bicylus breathing
35	–	851	1.8	CNH rocking

36	–	823	3.8	ring breathing
37	744	754	13.8	C-H wag
38	–	745	0	ring out of plane
39	684	690	4.2	B-N, B-O wag
40	–	639	0.8	in pl. ring deformation
41	–	622	0	In pl. ring deformation
42	–	582	0	ring out of plane
43	539	544	2.4	BNC bending
44	–	523	1	In pl. ring deformation
45	–	432	0.9	ring out of plane

[a]Obtained after irradiating neon matrix containing **5b** and doped with 3 % of C_2H_2 for 120 min; [b]Intensities are relative to the strongest band; [c]Computed absolute intensity: 719.1 km mol^{-1}; * could be observed only in Fig. S7b.

Cartesian Coordinates and Energies

Table S4. Cartesian coordinates and the energies of the optimized geometries calculated at the B2PLYP-D3/def2-TZVPP level of theory.

**3**			
-539.43473			
**C**	0.0565520	-0.9209950	0.6962620
**C**	0.0565520	-0.9209950	-0.6962620
**C**	-0.0308870	-2.0872130	-1.4254020
**C**	-0.1200570	-3.2783610	-0.6967170
**C**	-0.1200570	-3.2783610	0.6967170
**C**	-0.0308870	-2.0872130	1.4254020
**H**	-0.0291040	-2.0757150	-2.5046850
**H**	-0.1896760	-4.2163230	-1.2275860
**H**	-0.1896760	-4.2163230	1.2275860
**H**	-0.0291040	-2.0757150	2.5046850
**O**	0.1513820	0.3732190	1.1550950
**O**	0.1513820	0.3732190	-1.1550950
**B**	0.2175770	1.1594570	0.0000000
**C**	-0.2608510	3.5952110	-0.7464430
**C**	-0.2608510	3.5952110	0.7464430
**H**	0.3883880	4.2828160	1.2705520
**H**	0.3883880	4.2828160	-1.2705520
**N**	0.3967700	2.5500860	0.0000000
**H**	-1.1818490	3.3476720	1.2577170
**H**	-1.1818490	3.3476720	-1.2577170
**4a**			
-539.34404			
**C**	-1.0174970	-0.7684100	-0.0589980
**C**	-0.8033810	0.5740840	0.2392420
**C**	-1.8331040	1.4905170	0.2697560
**C**	-3.1109120	1.0042500	-0.0225210
**C**	-3.3254790	-0.3400440	-0.3258110
**C**	-2.2727350	-1.2595240	-0.3505520
**H**	-1.6551890	2.5287910	0.5046690
**H**	-3.9484620	1.6859770	-0.0133980
**H**	-4.3266930	-0.6803800	-0.5451090
**H**	-2.4279260	-2.3024460	-0.5806310
**O**	0.1764950	-1.4473310	-0.0006600
**O**	0.5384380	0.7805530	0.4688390
**B**	1.1291530	-0.4818750	0.3262060
**C**	3.4822100	0.8893790	-1.0430250
**C**	3.5970060	-0.0101530	0.1284790
**H**	3.8660740	0.5987900	1.0163500
**H**	4.3760200	1.3143890	-1.4718980

**N**	2.4947530	-0.7800280	0.5777720
**H**	2.5227610	1.2469450	-1.3811740
**H**	4.4682640	-0.6688690	0.0309020
**4b**			
-539.34634			
**C**	1.1207500	-0.7513290	0.0042180
**C**	0.9346760	0.6263560	-0.0727730
**C**	1.9939290	1.5097800	-0.0692420
**C**	3.2716260	0.9514950	0.0237980
**C**	3.4585810	-0.4288720	0.1056370
**C**	2.3770760	-1.3136030	0.0978400
**H**	1.8377090	2.5758330	-0.1318850
**H**	4.1320100	1.6041290	0.0331100
**H**	4.4612710	-0.8240040	0.1748630
**H**	2.5104770	-2.3828680	0.1572160
**O**	-0.0989160	-1.3810550	-0.0376060
**O**	-0.4118090	0.8983260	-0.1419430
**B**	-1.0393030	-0.3549850	-0.1243230
**C**	-4.7689170	0.1868990	-0.0320490
**C**	-3.3472950	0.3435940	0.3352460
**H**	-3.2403380	0.3148390	1.4347660
**H**	-5.5167170	0.8007430	0.4435320
**N**	-2.4398400	-0.5757460	-0.2480970
**H**	-5.0627200	-0.5368500	-0.7741480
**H**	-3.0030710	1.3692430	0.1011830
**5a**			
-539.46314			
**C**	-0.9311640	0.6958250	-0.1294140
**C**	-0.9311680	-0.6958290	-0.1294050
**C**	-2.0796930	-1.4257950	0.0888890
**C**	-3.2528110	-0.6968540	0.3116020
**C**	-3.2528070	0.6968680	0.3115930
**C**	-2.0796850	1.4258000	0.0888690
**H**	-2.0683800	-2.5050390	0.0854660
**H**	-4.1771230	-1.2275030	0.4861920
**H**	-4.1771160	1.2275250	0.4861750
**H**	-2.0683660	2.5050440	0.0854320
**O**	0.3452130	1.1533910	-0.3694030
**O**	0.3452060	-1.1534060	-0.3693880
**B**	1.1202220	-0.0000100	-0.5182220
**C**	3.6854260	0.0000200	1.2645760
**C**	3.5532690	-0.0000040	-0.2285480
**H**	4.4992980	-0.0000100	-0.7764910
**H**	4.2512110	0.8762160	1.5848300
**N**	2.4831800	-0.0000160	-0.8939760

**H**	4.2512090	-0.8761670	1.5848590
**H**	2.7143340	0.0000290	1.7538350
**5b**			
-539.46398			
**C**	-1.0631170	0.6957820	-0.0449620
**C**	-1.0631370	-0.6957930	-0.0449620
**C**	-2.2298520	-1.4259030	0.0284360
**C**	-3.4214370	-0.6968840	0.1028170
**C**	-3.4214180	0.6969390	0.1028150
**C**	-2.2298120	1.4259250	0.0284330
**H**	-2.2181640	-2.5051330	0.0270560
**H**	-4.3603590	-1.2274340	0.1608090
**H**	-4.3603250	1.2275150	0.1608050
**H**	-2.2180930	2.5051550	0.0270500
**O**	0.2334690	1.1529180	-0.1238090
**O**	0.2334360	-1.1529670	-0.1238170
**B**	1.0201650	-0.0000360	-0.1742930
**C**	4.8143400	0.0000260	0.0145880
**C**	3.3839670	0.0000220	0.4428360
**H**	3.2203870	0.0000860	1.5277270
**H**	5.3232300	-0.8757010	0.4208090
**N**	2.4209940	-0.0000550	-0.3719780
**H**	5.3231910	0.8758280	0.4206980
**H**	4.8899010	-0.0000410	-1.0686380
**$TS_{3\text{-}4a}$**			
-539.34235			
**C**	0.9757950	-0.7626190	-0.0007490
**C**	0.8118790	0.5990890	-0.2356420
**C**	1.8693700	1.4824570	-0.1897770
**C**	3.1222680	0.9409980	0.1131520
**C**	3.2863810	-0.4230920	0.3526760
**C**	2.2057340	-1.3084360	0.3001650
**H**	1.7306320	2.5364180	-0.3760750
**H**	3.9802260	1.5950070	0.1628230
**H**	4.2696530	-0.8060920	0.5824320
**H**	2.3218300	-2.3660830	0.4805890
**O**	-0.2377840	-1.3990980	-0.1270480
**O**	-0.5162820	0.8593890	-0.4939950
**B**	-1.1465820	-0.3878380	-0.4280660
**C**	-3.3911010	0.5422630	1.2856410
**C**	-3.5718340	0.1820890	-0.1512130
**H**	-3.5944040	1.0954730	-0.7705650
**H**	-4.0727460	0.1836710	2.0390910
**N**	-2.5225280	-0.6157420	-0.7321940
**H**	-2.5339970	1.1220670	1.5915690

**H**	-4.5290050	-0.3099100	-0.3213590
**TS_{4a-5a}**			
-539.34131			
**C**	1.0747220	-0.7923920	-0.0324820
**C**	0.7447150	0.5589350	0.0565190
**C**	1.7067620	1.5478110	0.0909600
**C**	3.0380720	1.1285330	0.0289370
**C**	3.3703050	-0.2241360	-0.0624130
**C**	2.3876740	-1.2162380	-0.0955250
**H**	1.4388930	2.5911840	0.1609740
**H**	3.8254080	1.8674820	0.0516230
**H**	4.4104990	-0.5113610	-0.1078470
**H**	2.6342700	-2.2647070	-0.1645930
**O**	-0.0647400	-1.5439610	-0.0396160
**O**	-0.6198810	0.6879840	0.0927590
**B**	-1.1305110	-0.6324350	0.0399570
**C**	-3.5520530	1.2464270	-0.3118400
**C**	-3.5344440	-0.1621920	0.0728750
**H**	-4.4952500	-0.6554580	-0.1007650
**H**	-4.5021140	1.7586530	-0.3021810
**N**	-2.4751920	-1.0384750	0.0812700
**H**	-2.6430480	1.8210640	-0.3840190
**H**	-3.6272950	0.1519690	1.1708040
**TS_{5a-5b}**			
-539.45912			
**C**	-0.0420240	-1.0574060	-0.6965390
**C**	-0.0420240	-1.0574060	0.6965390
**C**	0.0609700	-2.2229890	1.4244500
**C**	0.1663270	-3.4139650	0.6963480
**C**	0.1663270	-3.4139650	-0.6963480
**C**	0.0609700	-2.2229890	-1.4244500
**H**	0.0596970	-2.2112190	2.5037940
**H**	0.2490440	-4.3505370	1.2279130
**H**	0.2490440	-4.3505370	-1.2279130
**H**	0.0596970	-2.2112190	-2.5037940
**O**	-0.1559450	0.2324390	-1.1596180
**O**	-0.1559450	0.2324390	1.1596180
**B**	-0.2256280	1.0229120	0.0000000
**C**	0.7180150	4.5818010	0.0000000
**C**	-0.4409310	3.6314420	0.0000000
**H**	-1.4308330	4.1057540	0.0000000
**H**	0.6599290	5.2295350	-0.8761620
**N**	-0.3473020	2.3903330	0.0000000
**H**	1.6620900	4.0456220	0.0000000
**H**	0.6599290	5.2295350	0.8761620

**$TS_{4a\text{-}4b}$**			
-539.34399			
**C**	-0.8361840	0.6268920	-0.2179180
**C**	-0.9688060	-0.7466440	-0.0393480
**C**	-2.1823860	-1.3312210	0.2535840
**C**	-3.2809440	-0.4724450	0.3593470
**C**	-3.1484660	0.9035220	0.1773410
**C**	-1.9111640	1.4841420	-0.1185830
**H**	-2.2738140	-2.3979480	0.3894940
**H**	-4.2525110	-0.8862320	0.5855470
**H**	-4.0188710	1.5365480	0.2668850
**H**	-1.7970180	2.5478880	-0.2606780
**O**	0.4810920	0.9271970	-0.4863130
**O**	0.2574050	-1.3510600	-0.2060090
**B**	1.1396640	-0.3066390	-0.4786430
**C**	3.4572830	0.3299170	1.3496520
**C**	3.5643820	0.1768620	-0.1231950
**H**	4.5267280	-0.2730420	-0.4003570
**H**	4.1084280	1.0140850	1.8697930
**N**	2.5126340	-0.4828390	-0.8136750
**H**	2.7427630	-0.2444060	1.9174980
**H**	3.6072720	1.1809330	-0.5859560
**$TS_{3\text{-}4b}$**			
-539.34344			
**C**	1.1240050	-0.7591430	-0.0174710
**C**	0.8819050	0.6125630	-0.0099050
**C**	1.9060120	1.5372600	-0.0094140
**C**	3.2064990	1.0272970	-0.0082750
**C**	3.4498990	-0.3473100	-0.0105960
**C**	2.4045940	-1.2737780	-0.0143210
**H**	1.7061850	2.5978480	-0.0057720
**H**	4.0410300	1.7127460	-0.0046800
**H**	4.4692940	-0.7039240	-0.0111840
**H**	2.5816090	-2.3383340	-0.0195750
**O**	-0.0698760	-1.4342460	-0.0362470
**O**	-0.4742520	0.8324840	0.0019840
**B**	-1.0533160	-0.4442040	-0.0196500
**C**	-4.5186810	0.4789530	-0.4023880
**C**	-3.3763490	0.2072470	0.5061310
**H**	-3.7410130	-0.2507060	1.4354540
**H**	-5.5402690	0.3717170	-0.0755460
**N**	-2.4488860	-0.7201980	-0.0728150
**H**	-4.3248880	0.6382840	-1.4508390
**H**	-2.8974310	1.1503400	0.8116320
**$TS_{4b\text{-}5b}$**			

-539.34343			
**dC**	-1.1283570	-0.7589950	-0.0010050
**C**	-0.9142210	0.6185960	0.0078990
**C**	-1.9582680	1.5207350	0.0003430
**C**	-3.2491050	0.9852920	-0.0184620
**C**	-3.4643290	-0.3933720	-0.0285740
**C**	-2.3991460	-1.2976560	-0.0203810
**H**	-1.7808890	2.5854670	0.0072550
**H**	-4.0970870	1.6541770	-0.0257780
**H**	-4.4760600	-0.7711670	-0.0429910
**H**	-2.5543900	-2.3657210	-0.0276740
**O**	0.0746460	-1.4096080	0.0136380
**O**	0.4337310	0.8660510	0.0223770
**B**	1.0509730	-0.4037030	0.0267130
**C**	4.7339790	0.2136750	-0.1743090
**C**	3.3039600	0.3965830	0.0313620
**H**	2.9332210	1.3751480	-0.2991400
**H**	5.3785180	1.0777840	-0.1390150
**N**	2.4359680	-0.6689330	0.0406830
**H**	5.1645660	-0.7738520	-0.1079510
**H**	3.5113870	0.5585100	1.1475920

Table S4.

Cartesian coordinates of optimized geometries calculated at the CASPT2/def2-SV(P) level of theory and their respective energies calculated at CASPT2/def2-TZVPP//CASPT2/def2-SV(P).

**1[1]●**C_2H_4			
-538.58056			
**C**	-0.5816885	0.7381669	-0.6998725
**C**	-0.5816885	0.7381669	0.6998725
**C**	-1.5126913	0.0144413	1.4382785
**C**	-2.4547633	-0.7235775	0.7040179
**C**	-2.4547633	-0.7235775	-0.7040179
**C**	-1.5126913	0.0144413	-1.4382785
**H**	-1.5011803	0.0264968	2.5327390
**H**	-3.2086017	-1.3117303	1.2394854
**H**	-3.2086017	-1.3117303	-1.2394854
**H**	-1.5011803	0.0264968	-2.5327390
**O**	0.4423211	1.5297503	-1.1562672
**O**	0.4423211	1.5297503	1.1562672
**B**	1.0732972	1.9700567	0.0000000
**C**	1.8389671	-1.2853526	0.0000000
**C**	2.9316507	-0.4963298	0.0000000
**H**	3.4006959	-0.1648394	-0.9333849

**H**	1.3728218	-1.6226849	-0.9327779
**N**	2.2422576	2.8395794	0.0000000
**H**	1.3728218	-1.6226849	0.9327779
**H**	3.4006959	-0.1648394	0.9333849
**TS**			
-538.58166			
**C**	-0.7495585	0.5752979	-0.7007406
**C**	-0.7495585	0.5752979	0.7007406
**C**	-1.7821178	0.0027488	1.4384525
**C**	-2.8256194	-0.5818918	0.7042838
**C**	-2.8256194	-0.5818918	-0.7042838
**C**	-1.7821178	0.0027488	-1.4384525
**H**	-1.7699510	0.0139794	2.5328780
**H**	-3.6605122	-1.0482291	1.2394513
**H**	-3.6605122	-1.0482291	-1.2394513
**H**	-1.7699510	0.0139794	-2.5328780
**O**	0.3772733	1.2069362	-1.1550010
**O**	0.3772733	1.2069362	1.1550010
**B**	1.0791700	1.5491324	0.0000000
**C**	2.3895742	-1.1346950	0.0000000
**C**	3.3827925	-0.2094345	0.0000000
**H**	3.8335839	0.1466808	-0.9321782
**H**	1.9636412	-1.5170604	-0.9344480
**N**	2.3749841	2.1980734	0.0000000
**H**	1.9636412	-1.5170604	0.9344480
**H**	3.8335839	0.1466808	0.9321782
**4a’**			
-538.65338			
**C**	0.9627683	0.2886950	-0.6998789
**C**	0.9627683	0.2886950	0.6998789
**C**	2.0846237	-0.0730539	1.4367677
**C**	3.2249266	-0.4429210	0.7031018
**C**	3.2249266	-0.4429210	-0.7031018
**C**	2.0846237	-0.0730539	-1.4367677
**H**	2.0698634	-0.0659791	2.5312052
**H**	4.1342317	-0.7365929	1.2396813
**H**	4.1342317	-0.7365929	-1.2396813
**H**	2.0698634	-0.0659791	-2.5312052
**O**	-0.2705763	0.6888927	-1.1544879
**O**	-0.2705763	0.6888927	1.1544879
**B**	-1.0161941	0.9245657	0.0000000
**C**	-3.2260539	-0.9349967	0.0000000
**C**	-3.5040831	0.5433803	0.0000000
**H**	-4.1230774	0.8275850	0.8764141
**H**	-3.0290372	-1.4622292	0.9391140

**N**	-2.3611144	1.4182575	0.0000000
**H**	-3.0290372	-1.4622292	-0.9391140
**H**	-4.1230774	0.8275850	-0.8764141
**4a**			
-538.65885			
**C**	-1.0719775	-0.9415133	0.0434529
**C**	-0.8853233	0.3853419	0.4500734
**C**	-1.9404093	1.2871360	0.5300320
**C**	-3.2105040	0.8005623	0.1757548
**C**	-3.3978419	-0.5313476	-0.2346028
**C**	-2.3233965	-1.4342887	-0.3086844
**H**	-1.7790421	2.3208995	0.8516760
**H**	-4.0734541	1.4745629	0.2212538
**H**	-4.4039609	-0.8739748	-0.5016995
**H**	-2.4536636	-2.4745578	-0.6231725
**O**	0.1319965	-1.6001681	0.0690194
**O**	0.4440008	0.5922561	0.7257347
**B**	1.0555650	-0.6440444	0.4885705
**C**	3.3849589	0.5971103	-1.1000940
**C**	3.4763088	-0.1013733	0.2078092
**H**	3.5160362	0.7053161	0.9883500
**H**	4.2103863	1.2515225	-1.3996721
**N**	2.4370708	-0.9072032	0.7206615
**H**	2.4278589	0.7058074	-1.6226257
**H**	4.4553913	-0.6120439	0.3181631

References

1. W. Fraenk, T. Habereder, T. Klapoetke, H. Nöth and K. Polborn, *J. Chem. Soc., Dalton Trans.*, 1999, 4283-4286.
2. C. Lee, W. Yang and R. G. Parr, *Physical Review B*, 1988, **37**, 785-789.
3. A. D. Becke, *J. Chem. Phys.*, 1993, **98**, 5648-5652.
4. A. D. McLean and G. S. Chandler, *J. Chem. Phys.*, 1980, **72**, 5639-5648.
5. R. Krishnan, J. S. Binkley, R. Seeger and J. A. Pople, *J. Chem. Phys.*, 1980, **72**, 650-654.
6. S. Grimme and F. Neese, *J. Chem. Phys.*, 2007, **127**.
7. S. Grimme, J. Antony, S. Ehrlich and H. Krieg, *J. Chem. Phys.*, 2010, **132**.
8. H.-J. Werner, *Mol. Phys.*, 1996, **89**, 645-661.
9. P. Celani and H.-J. Werner, *J. Chem. Phys.*, 2000, **112**, 5546-5557.
10. F. Weigend and R. Ahlrichs, *Phys. Chem. Chem. Phys.*, 2005, **7**, 3297-3305.
11. M. J. Frisch, G. W. Trucks, H. B. Schlegel, G. E. Scuseria, M. A. Robb, J. R. Cheeseman, G. Scalmani, V. Barone, G. A. Petersson, H. Nakatsuji, X. Li, M. Caricato, A. V. Marenich, J. Bloino, B. G. Janesko, R. Gomperts, B. Mennucci, H. P. Hratchian, J. V. Ortiz, A. F. Izmaylov, J. L. Sonnenberg, Williams, F. Ding, F. Lipparini, F. Egidi, J. Goings, B. Peng, A. Petrone, T. Henderson, D. Ranasinghe, V. G. Zakrzewski, J. Gao, N. Rega, G. Zheng, W. Liang, M. Hada, M. Ehara, K. Toyota, R. Fukuda, J. Hasegawa, M. Ishida, T. Nakajima, Y. Honda, O. Kitao, H. Nakai, T. Vreven, K. Throssell, J. A. Montgomery Jr., J. E. Peralta, F. Ogliaro, M. J. Bearpark, J. J. Heyd, E. N. Brothers, K. N. Kudin, V. N. Staroverov, T. A. Keith, R. Kobayashi, J. Normand, K. Raghavachari, A. P. Rendell, J. C. Burant, S. S. Iyengar, J. Tomasi, M. Cossi, J. M. Millam, M. Klene, C. Adamo, R. Cammi, J. W. Ochterski, R. L.

Martin, K. Morokuma, O. Farkas, J. B. Foresman and D. J. Fox, *Gaussian 16 Rev. C.01, Wallingford, CT, 2016*.

12. H.-J. Werner, P. J. Knowles, F. R. Manby, J. A. Black, K. Doll, A. Heßelmann, D. Kats, A. Köhn, T. Korona, D. A. Kreplin, Q. Ma, T. F. Miller, III, A. Mitrushchenkov, K. A. Peterson, I. Polyak, G. Rauhut and M. Sibaev, *J. Chem. Phys.*, 2020, **152**.
